# Synthesis of mono-functionalized S-diazocines via intramolecular Baeyer–Mills reactions

**DOI:** 10.3762/bjoc.14.257

**Published:** 2018-11-07

**Authors:** Miriam Schehr, Daniel Hugenbusch, Tobias Moje, Christian Näther, Rainer Herges

**Affiliations:** 1 Otto Diels-Institute for Organic Chemistry, Christian-Albrechts-University, Otto-Hahn-Platz 4, 24118 Kiel, Germany; 2 Institute for Inorganic Chemistry, Christian-Albrechts-University, Max-Eyth-Str. 2, 24118 Kiel, Germany

**Keywords:** Baeyer–Mills reaction, bridged azobenzene, diazocine, reductive azo condensation, unsymmetrically functionalized S-diazocines

## Abstract

Herein we report a reliable method to synthesize mono-functionalized S-diazocines in reproducible yields via intramolecular Baeyer–Mills reactions. Diazocines exhibit excellent photoswitchable properties. As opposed to azobenzenes they are more stable in their *cis* configuration. Particularly in photopharmacology mono-functionalized diazocines should be potentially useful and superior to the frequently used azobenzenes because the sterically more demanding *cis* configuration should be inactive, and the slender *trans* configuration should fit in a tight binding pocket of a receptor. Hence, it should be possible to administer the stabile inactive compound and switch it on at the site of illness with visible light. To date only a limited number of diazocine derivatives have been published of which most are symmetrically functionalized. Using the Baeyer–Mills reaction for the synthesis of diazocines opens a novel and convenient access to unsymmetrically substituted diazocines.

## Introduction

Photopharmacology is a promising, rapidly evolving field in medicine aiming at the development of drugs whose biological activity can be controlled with light [1–4]. There are several reported pharmacologically active compounds containing azobenzenes as photoswitchable units [5–7]. In most cases these drugs are active in the more stable *trans* configuration and therefore have to be switched to the inactive form before administration. Unfortunately, the *cis* form of azobenzenes is not stable. In most cases, the *cis* isomer thermally isomerizes back to the active *trans* within several hours. Therefore, spatiotemporal control of activation with light is difficult to achieve. On this account, bridged azobenzenes (diazocines), would be more suitable as photoswitches in pharmacologically active compounds [8]. Besides their superior photophysical properties, diazocines are more stable in the *cis* configuration and could therefore be administered in their inactive form and switched on at the site of interest. However, applications of diazocines have been rather limited due to low yields and difficult syntheses. Moreover, the preparation of unsymmetrically functionalized diazocines, which are essential precursors for incorporation in a drug, are especially challenging because most synthetic approaches involve homocoupling steps for the synthesis of the bridge as well as for the formation of the azo function [9–16]. In Table 1 previous synthesis approaches are compared to the synthesis approach in the present study.

**Table 1:** Comparison of previous diazocine syntheses approaches compared to the present study discussed in this publication.

previous diazocine syntheses	present synthesis approach

symmetrical approach:	
Duval [9], Herges [12–13,15], Woolley [11] and Xie [14]	
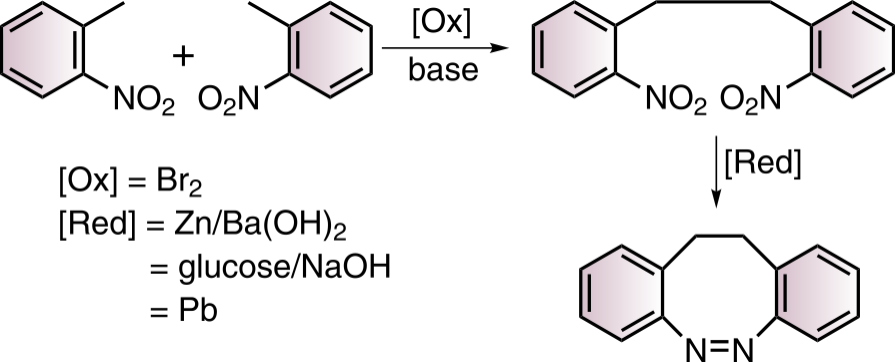	
unsymmetrical approach:	
Yan [10,16]	
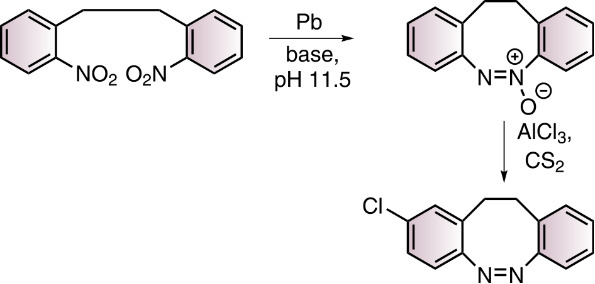	
Herges [17]	
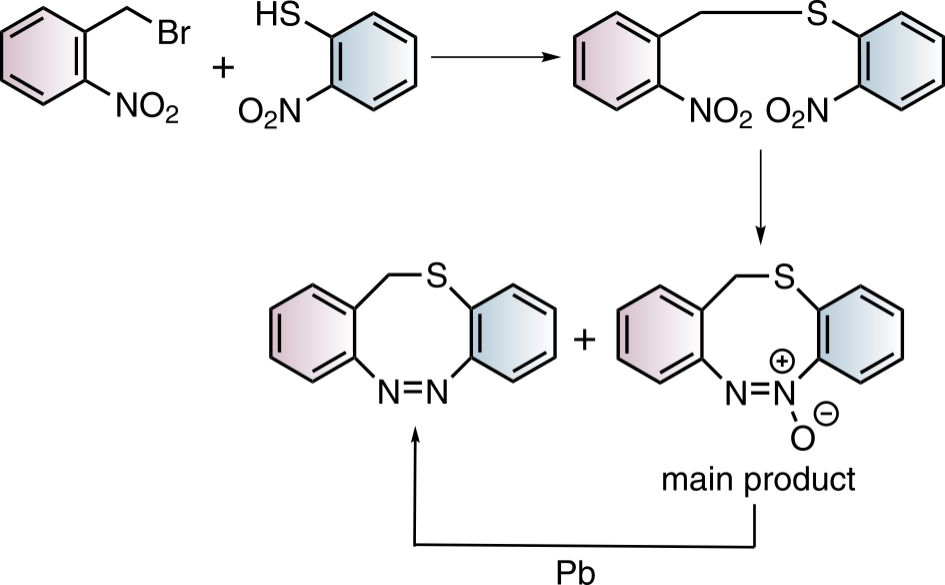	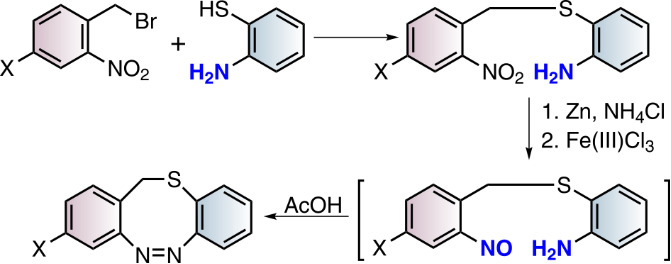

Recently, we reported the synthesis of a diazocine where the –CH_2_–CH_2_– bridge is replaced by a –CH_2_–S– bridge (S-diazocine) using an ultrasound-supported reductive azocoupling with lead [17]. In this reaction a small amount of S-diazocine is directly formed and a larger fraction of the azoxy compound is obtained, which can be reduced in a second synthetic step using lead in a ball mill. However, these reaction conditions are not suitable for functionalized S-diazocines due to poor yields and low reproducibility. Using halogenated precursors for the reductive azocoupling with lead a large amount of byproduct (details see Supporting Information File 1) is formed, which lowers the overall yield of S-diazocines **1**–**3** considerably. Further problems arise from the difficult separation of the azoxy compound from other byproducts. The yields of the azoxy reduction in the ball mill are varying between 18–46%. S-Diazocines functionalized with a carboxylic acid **4** or a benzyl alcohol **5** could not be obtained using the reductive azo coupling. For the benzyl alcohol **5** a variety of different, unidentified byproducts formed and in case of the carboxylic acid **4** only the starting material was retrieved. We now present a reliable one-pot-like synthesis of mono-functionalized S-diazocines in reproducible yields via intramolecular Baeyer–Mills reaction.

## Results and Discussion

The Baeyer–Mills reaction is a quite common method, particularly to synthesize unsymmetrically substituted azobenzenes, albeit not widely used to prepare diazocine derivatives [18–19].

**Figure 1:**
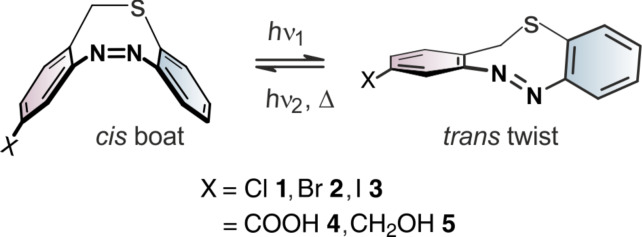
*Cis*–*trans* isomerization of mono-functionalized S-diazocines **1**–**5**.

Towards the synthesis of S-diazocines (Figure 1), two general approaches could be derived from the well investigated azobenzene preparation methods: a) Reductive coupling of two nitro groups or b) oxidative coupling of two amino groups [20]. To cleanly form N=N bonds by reduction of dinitro compounds, strongly basic conditions have to be applied. Unfortunately, the benzylic position of the bridge is deprotonated under these conditions, leading to a plethora of side products. Formation of the N=N double bond by oxidation of diamines [21–23] cannot be applied either, because the sulfur bridge is oxidized to the sulfoxide or sulfone. Hence, neither reductive nor oxidative conditions are compatible with the –CH_2_-S– bridge. An alternative strategy, that applies weakly acidic conditions is the condensation of aryl nitroso compounds with anilines. This Baeyer–Mills reaction was discovered by Baeyer in 1874 [24] and further investigated by Mills [25] and Bamberger [26].

**Scheme 1:**
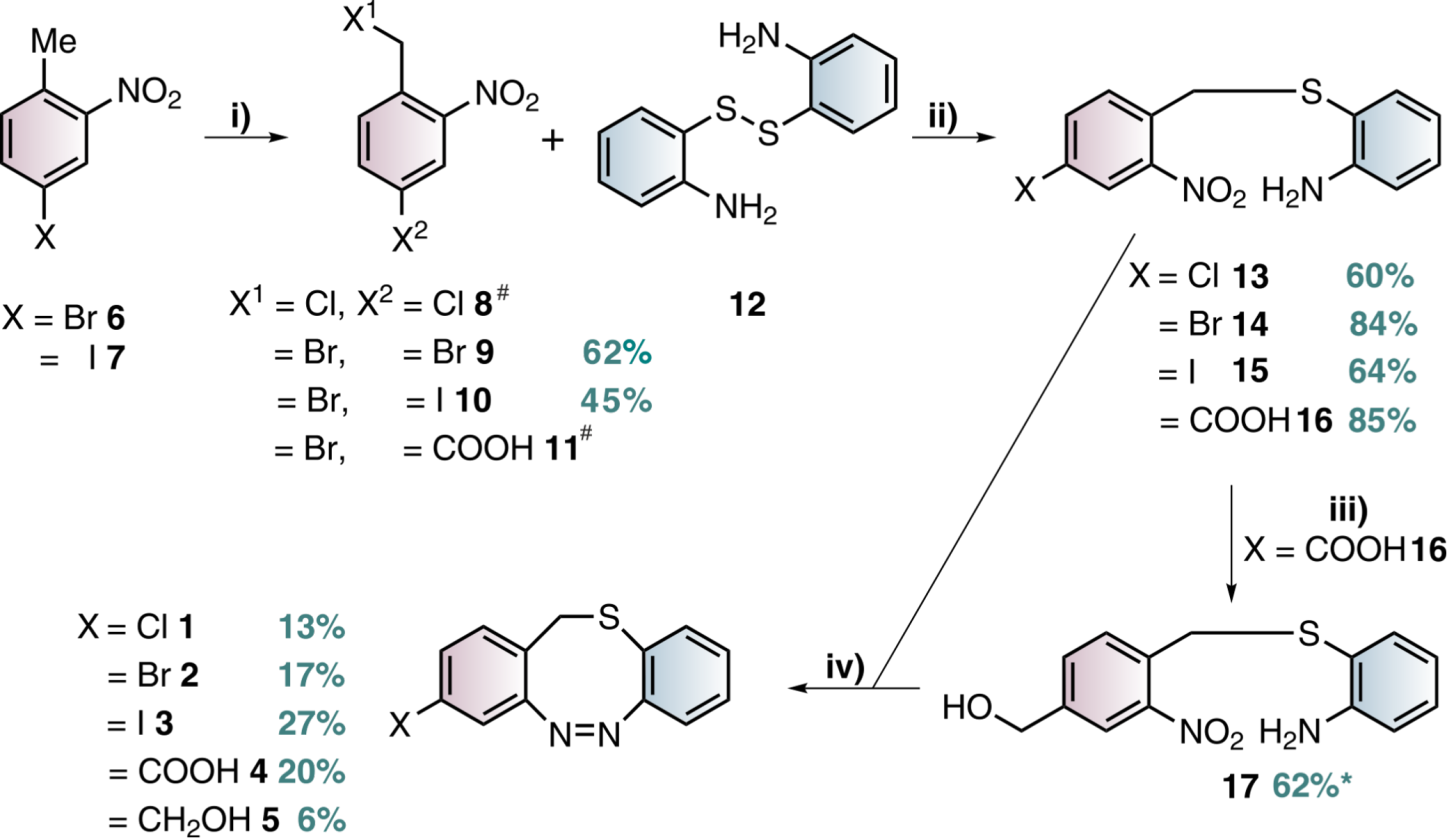
Reaction conditions: i) MeCN, AIBN, NBS; ii) NaBH_4_, THF; ^#^commercially available iii) BH_3_·THF complex, THF, *product **17** is not stable; iv) 1. Zinc powder, ammonium chloride, ethanol, 2. Fe(III)Cl_3_ hexahydrate, H_2_O/ethanol, acetic acid.

The S-diazocines **1**–**5** were obtained using a 2–3 step synthesis (Scheme 1). To synthesize the nitro and amino-substituted precursors **13**–**16**, disulfide **12** was reduced with sodium borohydride and in situ reacted with benzyl bromides **8**–**11**, which were commercially available or synthesized by radical bromination. The S-diazocines precursors **13**–**16** were then subjected to the intramolecular Baeyer–Mills reaction yielding the S-diazocines **1**–**4**. In the first step the nitro group was reduced with zinc powder to the hydroxylamine and subsequently in situ oxidized to the nitroso compound using iron(III) chloride.

After addition of acetic acid the reaction was allowed to proceed for several hours at room temperature to form the azo bond. For the benzyl alcohol-functionalized S-diazocine **5**, the carboxylic acid of the precursor **16** was reduced using a borane tetrahydrofuran complex and immediately reacted in the Baeyer–Mills reaction without further purification. In Table 2 the yields using the Baeyer–Mills reaction are compared with the yields using the reductive azo condensation with lead [17].

**Table 2:** Comparison of the obtained S-diazocine yields using the reductive lead method or the Baeyer–Mills reaction.

X	Cl	Br	I	COOH	CH_2_OH

molecule	**1**	**2**	**3**	**4**	**5**

yield lead method	5%	13%	<2%	–	–
yield Baeyer–Mills reaction	13%	17%	27%	20%	6%

Compared to the reductive azo condensation, the yields obtained using the Baeyer–Mills reaction are higher, more reproducible and applicable for an enlarged number of functional groups. Using this method, five different mono-functionalized S-diazocines **1**–**5** could be synthesized, which are suitable starting materials for further functionalization such as cross coupling, peptide coupling or further functionalization.

The photoswitchable properties of the S-diazocines **1**–**5** were investigated using UV–vis and NMR spectroscopic methods. The photostationary states (PSS), half-lives (*t*_1/2_) and absorption maxima (λ_max_) were recorded in acetone and are summarized in Table 3.

**Table 3:** Photostationary states, absorption maxima and half-lives of S-diazocines **1**–**5** determined with UV–vis and NMR spectroscopy in acetone.

molecule	X	PSS (405 nm)	λ_max_ (*cis*)	λ_max_ (*trans*)	*t*_1/2_ (NMR)

**1**	Cl	64%	392 nm	516 nm	3.6 d
**2**	Br	60%	389 nm	516 nm	2.8 d
**3**	I	51%	393 nm	515 nm	3.3 d
**4**	COOH	67%	393 nm	516 nm	6.7 d
**5**	CH_2_OH	65%	393 nm	508 nm	24.8 h

The photostationary states (PSS) upon irradiation with 405 nm are between 51% and 67% *trans*, and thus similar to the PSS of the parent system (70%) [17]. The absorption maxima of the nπ* excitation of the *cis* isomers are between 389 nm and 393 nm. For the *trans* isomers the nπ* absorption maxima are in a range between 508 nm to 516 nm (for UV–vis spectra measured in acetone see Supporting Information File 1). The half-lives vary from 24.8 h to 6.7 d.

**Figure 2:**
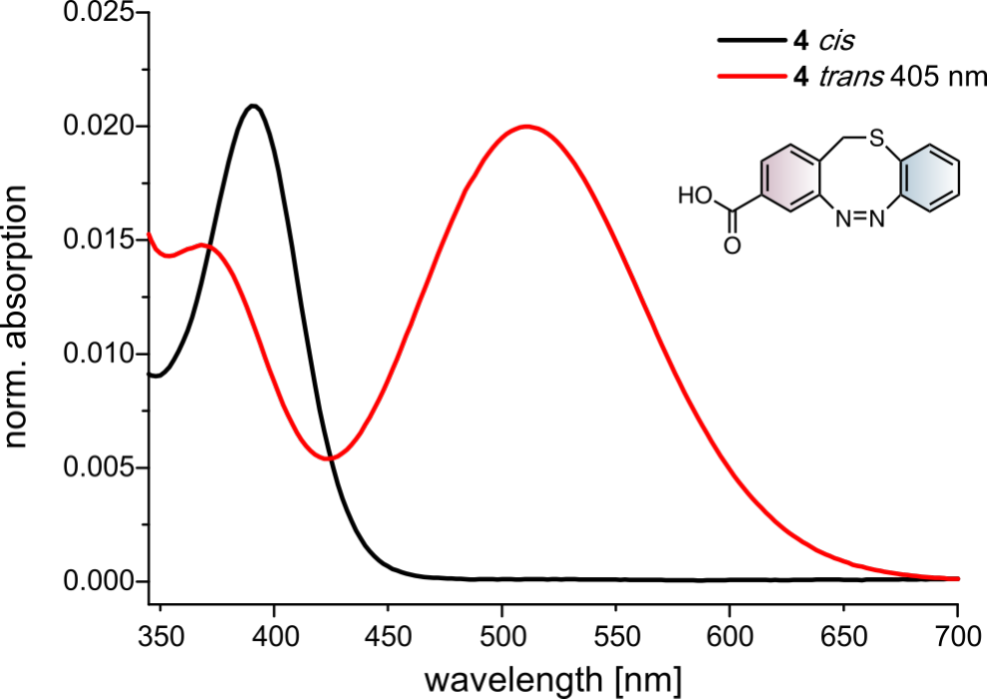
UV spectra of the S-diazocine **4** in *cis* (black) and in *trans* (red) configuration after irradiation with 405 nm and separation by chromatography measured in a mixture of acetonitrile and water.

For the carboxylic acid functionalized S-diazocine **4** the two isomers generated after irradiation with 405 nm could be separated by chromatography and the UV–vis spectra of the pure *cis* and *trans* isomers could be measured in an acetonitrile/water mixture. Figure 2 shows the UV spectra of the pure *cis* and *trans* isomers of **4**.

The nπ* band of the *cis* isomer displays an absorption maxima at λ_max_ = 392 nm. Upon irradiation with 405 nm to the *trans* isomer the nπ* band is shifted bathochromically to λ_max_ = 512 nm. The *trans* isomer exhibits also an additional absorption maximum in the same area as the *cis* isomer at 369 nm, which is the reason for the low *cis*-to-*trans* conversion when compared to the C-diazocine (>90% *trans*) [8]. The back isomerization is initiated quantitatively with light within the range of 500–660 nm. Hence, the *trans*-to-*cis* isomerization can be performed with red light which is important for medical applications, because blood-supported tissue is transparent in the far red region [27].

For the iodo-functionalized S-diazocine **3** single crystals could be obtained, which were characterized by single crystal X-ray diffraction. The structure of **3** is the first crystal structure of a S-diazocine reported in the Cambridge Structural Database (CSD; version 5.39; Feb. 2018) [28].

**Figure 3:**
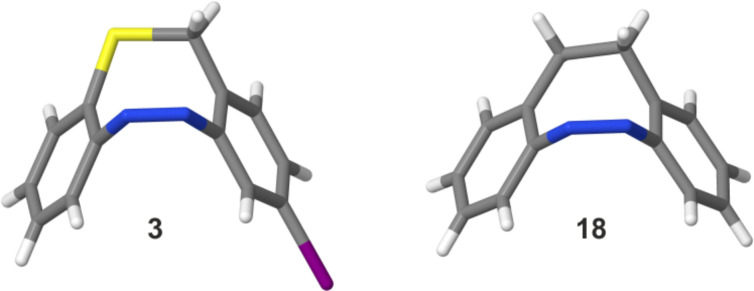
Left: crystal structure of the iodo-functionalized S-diazocine **3**. Right: crystal structure of the unfunctionalized C-diazocine **18** [8].

This compound crystallizes in the monoclinic space group *P*2_1_/*c* with *Z* = 4 and all atoms in general positions (specification see Supporting Information File 1). The molecule exhibits a *cis* boat conformation with an dihedral angle of 72.24(8)° between both phenyl rings (Figure 3). Each of these rings is planar with maximum deviations from the mean plane calculated through the C atoms of 0.014(2) and 0.009(2) Å. The N=N bond lengths of the azo group of 1.251(4) Å corresponds to literature values and the CNNC torsion angle amounts to 3.7(4)°, which proves that this unit is nearly coplanar. In contrast, the CCSC fragment is twisted by 22.9(3)°. The structure of the title compound **3** can be compared with the structure of 5,6-dihydrobenzo[*c*,*g*][1,2]-diazocine (**18**) already reported in the literature [8]. In this compound the CNNC fragment is more planar with an torsion angle of 0.7(2)°. The dihedral angle between the phenyl rings of 85.00(6)° is larger compared to the S-diazocine **3**. The ethylene group in the 8-membered ring is disordered in two orientations with torsion angles of 47.9(1) and 37.7(1)°, which means that the overall molecule is more distorted in the C-diazocine than in the sulfur analogue.

## Conclusion

The Baeyer–Mills reaction as the key step in our approach provides an easy one-pot-like synthesis for mono-functionalized S-diazocines. Using this method, five different mono-functionalized S-diazocines **1**–**5** have been synthesized and were investigated regarding their photochemical properties. The S-diazocines **1**–**5** are potential precursors for the incorporation in photopharmacophores using cross-coupling, peptide coupling or further functionalization methods such as nucleophilic substitution reaction of the benzyl alcohol **5**.

The yield determining steps prior to the Baeyer–Mills reaction are the formation of the hydroxylamine with zinc and the oxidation from the hydroxylamine to the nitroso compound. These reaction steps are difficult to monitor and furthermore the S-diazocine yield depends on the nature of substituents. However, the overall yields and the reliability of the formation of the azo group are superior to previously reported methods. All S-diazocines **1**–**5** are thermodynamically more stable in their *cis* configuration. Irradiation with violet light (405 nm) converts the *cis* into the *trans* isomer with 50–70% yield. Back-reaction with green to red light (500–660 nm) is quantitative. Thermal half-lives of the metastable *trans* isomer are between 1–7 days. These properties are ideal for applications in photopharmacology where the more bulky *cis* state is the inactive form and the *trans* isomer is the active configuration.

## Supporting Information

CCDC-1857720 (**3**) contains the supplementary crystallographic data for this paper. These data can be obtained free of charge from the Cambridge Crystallographic Data Centre via http://www.ccdc.cam.ac.uk/data_request/cif.

File 1 Analytical equipment, experimental procedures, spectral data and crystallographic data.
