# Supplementary material for: Synthesis of mono-functionalized S-diazocines via intramolecular Baeyer–Mills reactions
Source: Beilstein J Org Chem. 2018 Nov 7;14:2799–804. doi: 10.3762/bjoc.14.257 (PMC6244113; doi:10.3762/bjoc.14.257)
Supplement: File 1 — Analytical equipment, experimental procedures, spectral data and crystallographic data. [file Beilstein_J_Org_Chem-14-2799-s001.pdf]

Supporting Information

for

Synthesis of mono-functionalized S-diazocines via intramolecular Baeyer–Mills reactions

Miriam Schehr[1], Daniel Hugenbusch[1], Tobias Moje[1], Christian Näther[2] and Rainer Herges*[1]

Address: [1]Otto Diels-Institute for Organic Chemistry, Christian-Albrechts-University, Otto-Hahn-Platz 4, 24118 Kiel, Germany and [2]Institute for Inorganic Chemistry, Christian-Albrechts-University, Max-Eyth-Str. 2, 24118 Kiel, Germany

Email: Rainer Herges* - rherges@oc.uni-kiel.de

* Corresponding author

Analytical equipment, experimental procedures, spectral data and crystallographic data

Table of contents

I. Analytical equipment ··· S2–S3

II. Syntheses ··· S4–S27

III. Isolated byproduct reductive azo coupling ··· S28–S29

IV. UV–vis switching experiments ··· S30

V. Crystallographic data compound **3** ··· S31–S33

I. Analytical equipment

NMR spectroscopy

NMR spectra were measured in deuterated solvents (Deutero). To reference the NMR spectra the following solvent signals were used [1]:

solvent	degree of deuteration	^{1}H signal	^{13}C signal
acetone-d_6	99.8 %	2.05 (quintet)	29.84 (septet)
chloroform-d_1	99.8 %	7.26 (s)	77.16 (triplet)
DMSO-d_6	99.8 %	2.50 (s)	39.52 (septet)

NMR measurements were performed with a Bruker DRX 500 (^{1}H NMR: 500 MHz, ^{13}C NMR: 125 MHz, ^{19}F NMR: 470 MHz) and a Bruker AV 600 (^{1}H NMR: 600 MHz, ^{13}C NMR: 150 MHz) spectrometer.
Signals were abbreviated with s, bs, d, t, m and m_c for singlet, broad singlet, doublet, triplet, multiplet and centered multiplet.

Melting point

Melting points were measured with a Melting Point B-560 (Büchi) in melting point tubes.

Mass spectrometry

The high resolution (HR-EI) mass spectra were measured with an AccuTOF GCv 4G (Joel) with ionization energy of 70 eV and the high resolution (HR-ESI) mass spectra were measured with a Thermo Fischer Q Exactive Plus MS, Hybrid Quadrupol-Orbitrap.

IR spectroscopy

Infrared spectra were measured on a Perkin-Elmer 1600 Series FT-IR spectrometer with an A531-G Golden-Gate-Diamond-ATR-unit. Signals were abbreviated with w, m, s, vs for weak, medium, strong and very strong signal intensity.

UV–vis spectroscopy

UV-Vis spectra were measured with a Lambda 650 spectrometer (Perkin-Elmer). Quartz cuvettes of 1 cm optical path length were used.

Chromatography stationary phases

For column chromatography purifications silica gel (Merck, particle size 0.040–0.063 mm) was used. Flash column chromatography purfications were performed on a Biotage® type Isolera one with Biotage® Ultra cartridges (Biotage®, HP-Sphere™, particle diameter: 25 µm, cartridges sizes: 10 g, 25 g, 50 g and 100 g) were used. R_f values were determined by thin layer chromatography on Polygram® SilG/UV254 (Macherey Nagel, 0.2 mm particle size).

HPLC measurements

Separation of the *cis* and *trans* isomers of compound **4** was performed using a HPLC (Agilent 1100 Series) with diode array UV/vis detector (Agilent 1200 Series). As stationary phase a reversed phase column type ZORBAX Eclipse XBD-C8 (Agilent, 150 × 46 mm, particle size: 5 µM) with an acetonitrile/water gradient as eluent was used.

Light sources

UV–vis and ^{1}H NMR:

The irradiation of the samples was performed with LEDs with a wavelength of 405 nm from SAHLMANN PHOTOCHEMICAL SOLUTIONS with followed specifications:

- 405 nm: 3 x NCSU276A-U405, FWHM = 13 nm, *P* (opt) = 3 x 1300 mW

II. Syntheses

II.1. Synthesis of 4-bromo-1-(bromomethyl)-2-nitrobenzene (9)

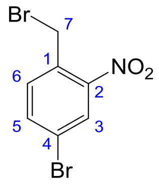

4-Bromo-1-methyl-2-nitrobenzene (**6**) (2.00 g, 9.26 mmol), NBS (1.90 g, 10.7 mmol) and AIBN (160 mg, 0.97 mmol) were dissolved in 15 mL dry acetonitrile under nitrogen atmosphere. The mixture was refluxed under irradiation with light and after 24 h additional AIBN (160 mg, 0.97 mmol) was added. After 48 h the solvent was removed in vacuo and the crude product was purified by flash column chromatography (cyclohexane/chloroform, chloroform: 5% → 29%) to yield a yellowish solid (1.69 g, 5.73 mmol, 62%).

melting point: 73 °C.

R_f : 0.44 (cyclohexane/chloroform, 4:1).

^{1}H NMR (500 MHz, $CDCl_3$): δ = 8.19 (d, ^4J = 2.01 Hz, 1 H, *H*-3), 7.74 (dd, ^3J = 8.30 Hz, ^4J = 2.01 Hz, 1 H, *H*-5), 7.46 (d, ^3J = 8.30 Hz, 1 H, *H*-6), 4.77 (s, 2 H, *H*-7) ppm.

^{13}C NMR (125 MHz, $CDCl_3$): δ = 148.2 (*C*-2), 136.7 (*C*-5), 133.8 (*C*-1), 131.8 (*C*-6), 128.5 (*C*-3), 122.9 (*C*-4), 27.9 (*C*-7) ppm.

IR (ATR): ῦ = 3085 (w), 3053 (8) , 2864 (w), 1600 (w), 1562 (w), 1524 (vs), 1480 (m), 1435 (m), 1203 (w), 1225 (w), 1223 (w), 1126 (w), 1088 (m), 972 (w), 890 (m), 875 (m), 840 (m), 805 (s), 763 (m), 686 (s), 613 (s), 638 (w), 567 (m) cm^{-1}.

MS (EI, 70 eV): m/z (%) = 292 (6) $[M]^+$, 213 (100) $[M-Br]^+$.

MS (EI, HR, 70 eV): $C_7H_5Br_2NO_2$, m/z = calc.: 292.8687, found: 292.8689.

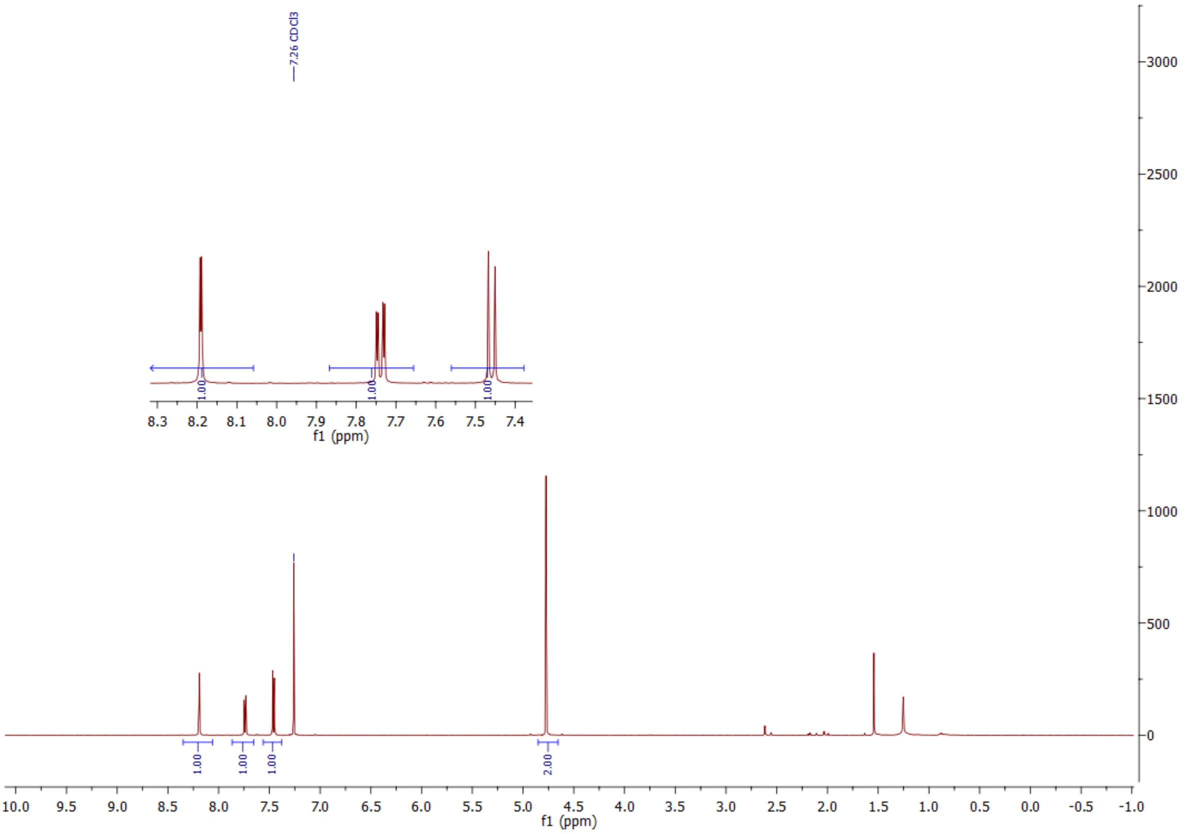

Figure S1: ^{1}H NMR spectrum of compound **9** measured in deuterated chloroform.

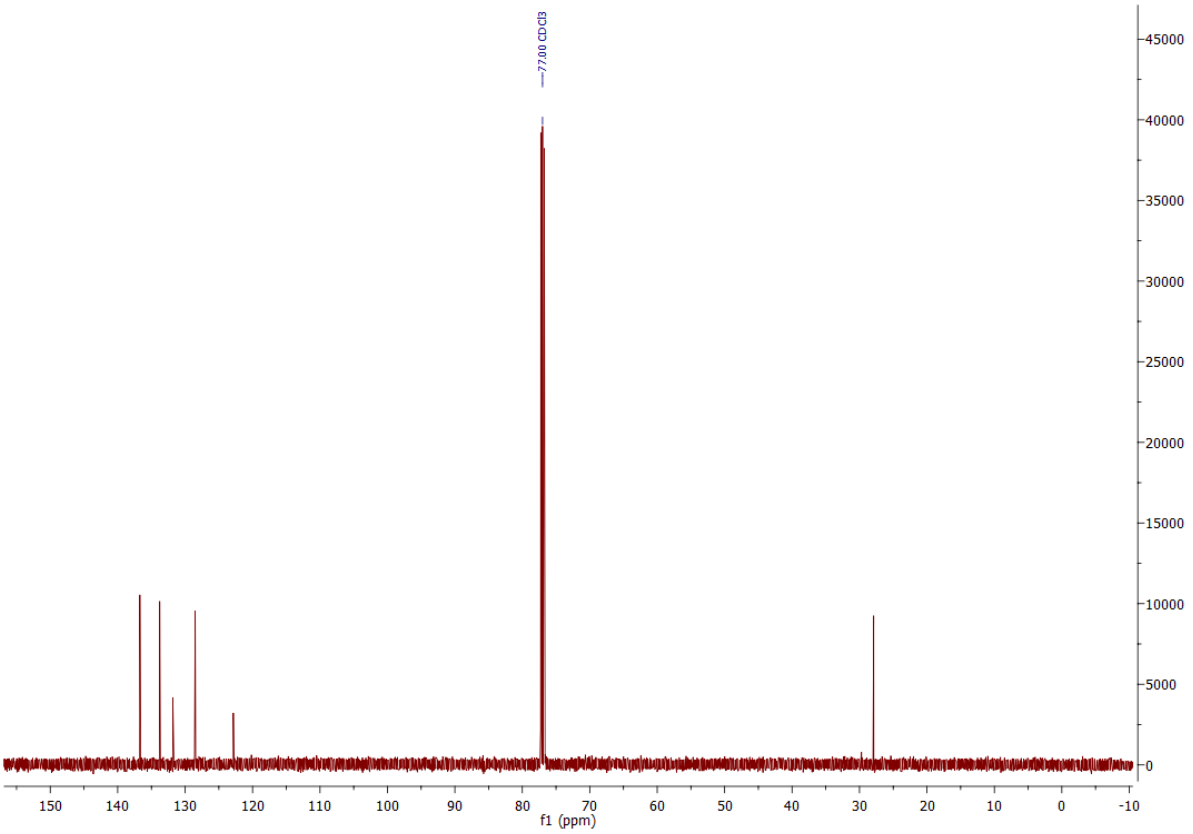

Figure S2: ^{13}C NMR spectrum of compound **9** measured in deuterated chloroform.

II.2. Synthesis of 4-iodo-1-(bromomethyl)-2-nitrobenzene (10)

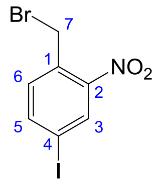

4-Iodo-1-methyl-2-nitrobenzene (**7**) (3.60 g, 13.7 mmol), NBS (2.90 g, 16.3 mmol) and AIBN (70 mg, 0.43 mmol) were dissolved in 15 mL dry acetonitrile under nitrogen atmosphere. The mixture was refluxed under irradiation with light and after 24 h additional AIBN (150 mg, 0.91 mmol) was added. After 48 h the solvent was removed in vacuo. The crude product was filtered over silica gel with DCM and refluxed in 20 mL petroleum ether for 1 h. After filtration a beige solid was obtained. The yield was determined via ^{1}H NMR which contains mostly a mixture of starting material **7** and desired product **10** (2.11 g, 6.17 mmol, 45%).

^{1}H NMR (500 MHz, $CDCl_3$): δ = 8.35 (d, ^{4}J = 1.77 Hz, 1 H, *H*-3), 7.93 (dd, ^{3}J = 8.15 Hz, ^{4}J = 1.7 Hz, 1 H, *H*-5), 7.30 (d, ^{3}J = 8.15 Hz, *H*-6), 4.76 (s, 2 H, *H*-7) ppm.

^{13}C NMR (125 MHz, $CDCl_3$): δ = 148.1 (*C*-4), 134.1 (*C*-3), 132.6 (*C*-1), 142.6 (*C*-5), 133.8 (*C*-6), 28.0 (*C*-7) ppm.

MS (EI, 70 eV): m/z (%) = 341 (11) $[M]^+$, 261 (100) $[M\text{-}Br]^+$, 246 (78) $[M\text{-}BrO]^+$.

MS (HR-ESI): m/z (%) = $[C_7H_5BrINO_2]^+$, m/z = calc.: 340.8548, found.: 340.8545.

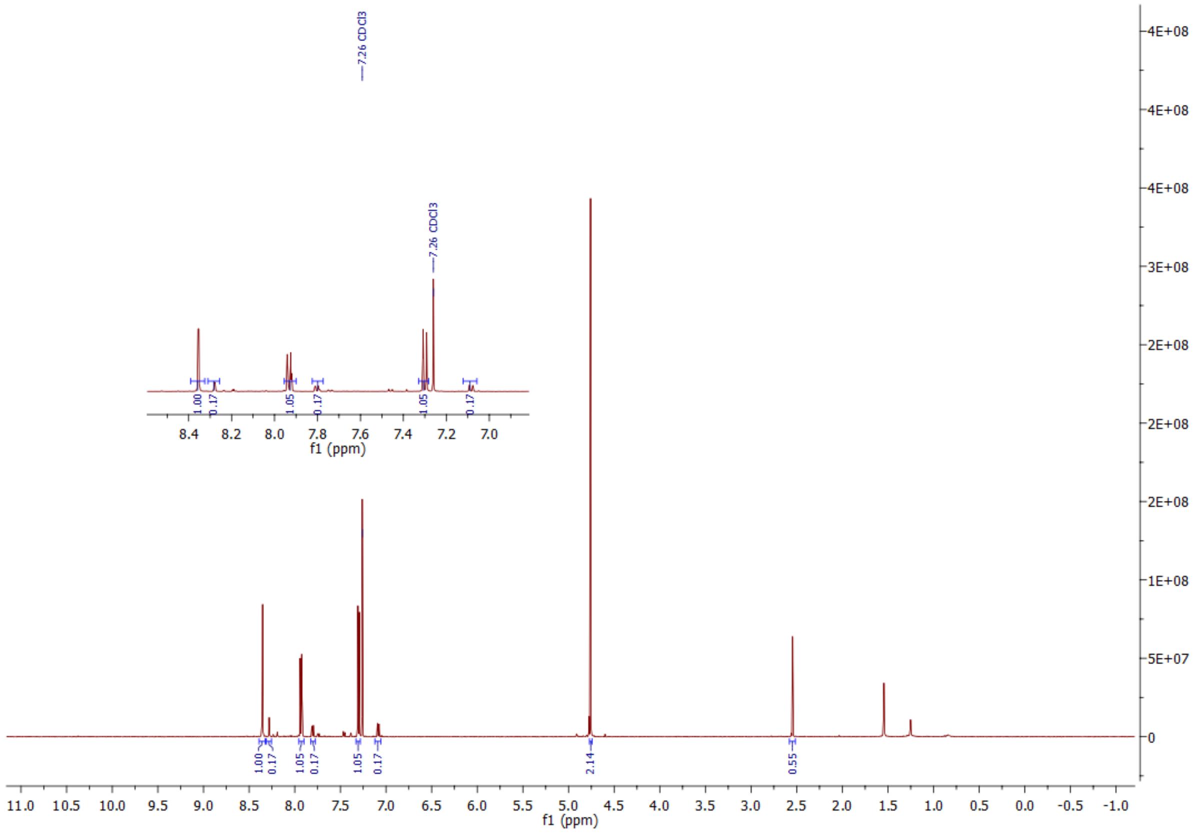

Figure S3: ^{1}H NMR spectrum of compound **10** measured in deuterated chloroform.

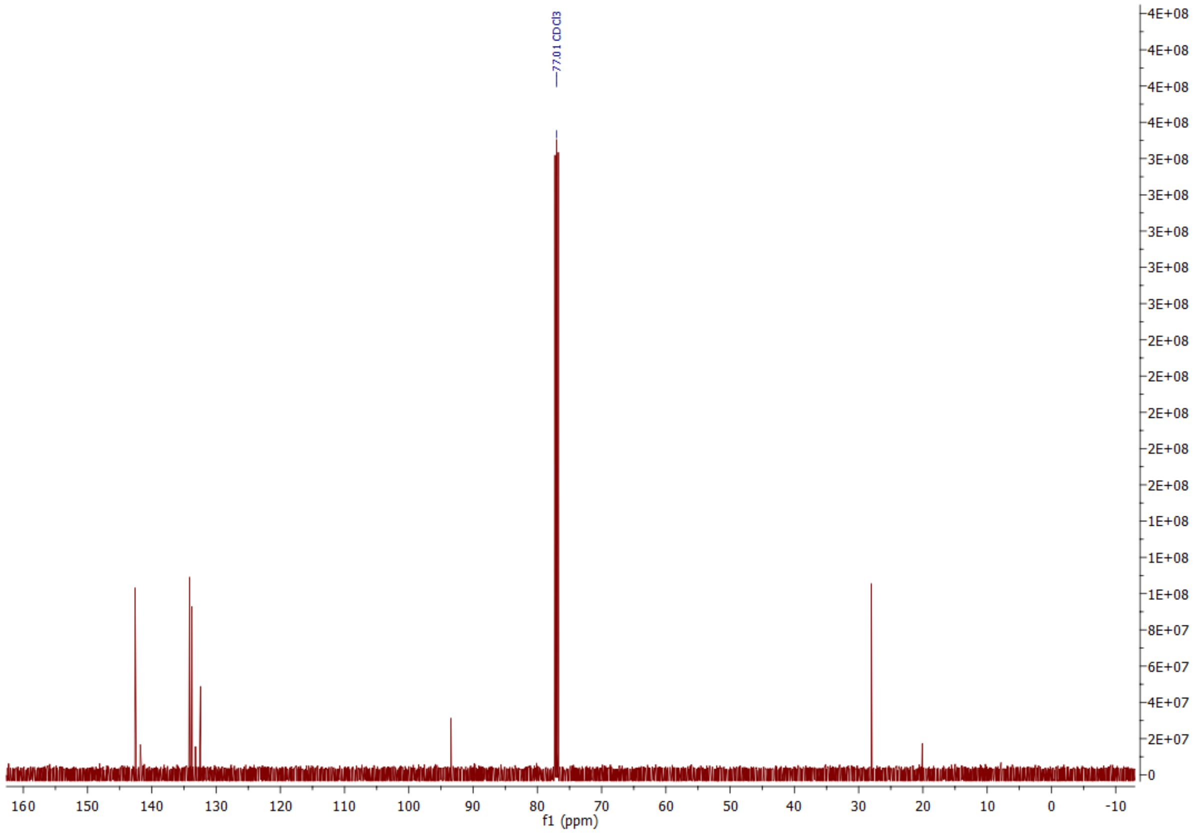

Figure S4: ^{13}C NMR spectrum of compound **10** measured in deuterated chloroform.

II.3. General method for the synthesis of the substituted thioether derivatives 13-16:

2,2'-Disulfanediyldianiline (**12**) (0.55 equiv) was dissolved in dry THF under nitrogen atmosphere. Sodium borohydride (1.5 equiv) was added and the solution was heated at 60 °C for 1 h. In the process the initially yellow solution turns to a milky white. The solution was cooled to 45 °C and the substituted benzylchoride/bromide **8**, **9**, **10** or **11** (1 equiv) was added. After stirring for 1 h at 45 °C the solution was cooled to room temperature and diluted with water. The mixture was extracted with DCM, the combined organic layers were dried over magnesium sulfate, filtered and the solvent was removed in vacuo. The crude product was purified on silica using flash column chromatography.

II.3.1. 2-((4-Chloro-2-nitrobenzyl)thio)aniline (13)

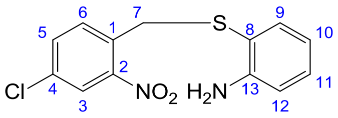

2,2'-Disulfanediyldianiline (**12**) (1.62 g, 6.51 mmol), sodium borohydride (671 mg, 17.7 mmol), 4-chloro-1-(chloromethyl)-2-nitrobenzene (**8**) (2.44 g, 11.8 mmol) in 40 mL dry THF.

Flash chromatography: cyclohexane/ethyl acetate, ethyl acetate: 12% → 100%.

An orange solid was obtained (2.10 g, 7.12 mmol, 60%).

melting point: 86 °C.

R*f* : 0.62 (cyclohexane/ethyl acetate, 1:1).

^{1}H NMR (500 MHz, $CDCl_3$): δ = 7.96 (d, ^4J = 2.2 Hz, 1 H, *H*-3), 7.34 (dd, ^3J = 8.3 Hz, ^4J = 2.2 Hz, 1 H, *H*-5), 7.13 (m_c, 1 H, *H*-10), 7.04 (dd, ^3J = 7.8 Hz, ^4J = 1.5 Hz, 1 H, *H*-9), 6.91 (d, ^3J = 8.3 Hz, 1 H, *H*-6), 6.70 (dd, ^3J = 8.0 Hz, ^4J = 1.3 Hz, 1 H, *H*-12), 6.57 (dt, ^3J = 7.5 Hz, ^4J = 1.3 Hz, 1 H, *H*-11), 4.31 (bs, 1 H, 2 H, NH_2), 4.21 (s, 2 H, *H*-7) ppm.

^{13}C NMR (125 MHz, $CDCl_3$): δ = 148.9 (*C*-8), 148.5 (*C*-4), 137.1 (*C*-9), 133.7 (*C*-1), 133.3 (*C*-5), 132.7 (*C*-6), 132.5 (*C*-13), 130.9 (*C*-10), 125.3 (*C*-3), 118.5 (*C*-11), 115.4 (*C*-2), 114.9 (*C*-12), 35.6 (*C*-7) ppm.

IR (ATR): ṽ = 3474 (m), 3376 (m), 1611 (m), 1566 (m), 1522 (s), 1478 (s), 1451 (m), 1432 (m), 1339 (vs), 1306 (m), 1255 (w), 1216 (w), 1160 (m), 1123 (m), 884 (m), 839 (m), 807 (m), 758 (vs), 735 (m), 714 (m), 656 (m), 530 (m) cm^{-1}.

MS (EI, 70 eV): m/z (%) = 294 (66) $[M]^+$, 124 (100) $[M-C_7H_6ClNO_2]^+$.

MS (EI, HR, 70 eV): $[C_{13}H_{11}ClN_2O_2S]$, m/z = calc.: 294.0230, found: 294.0231.

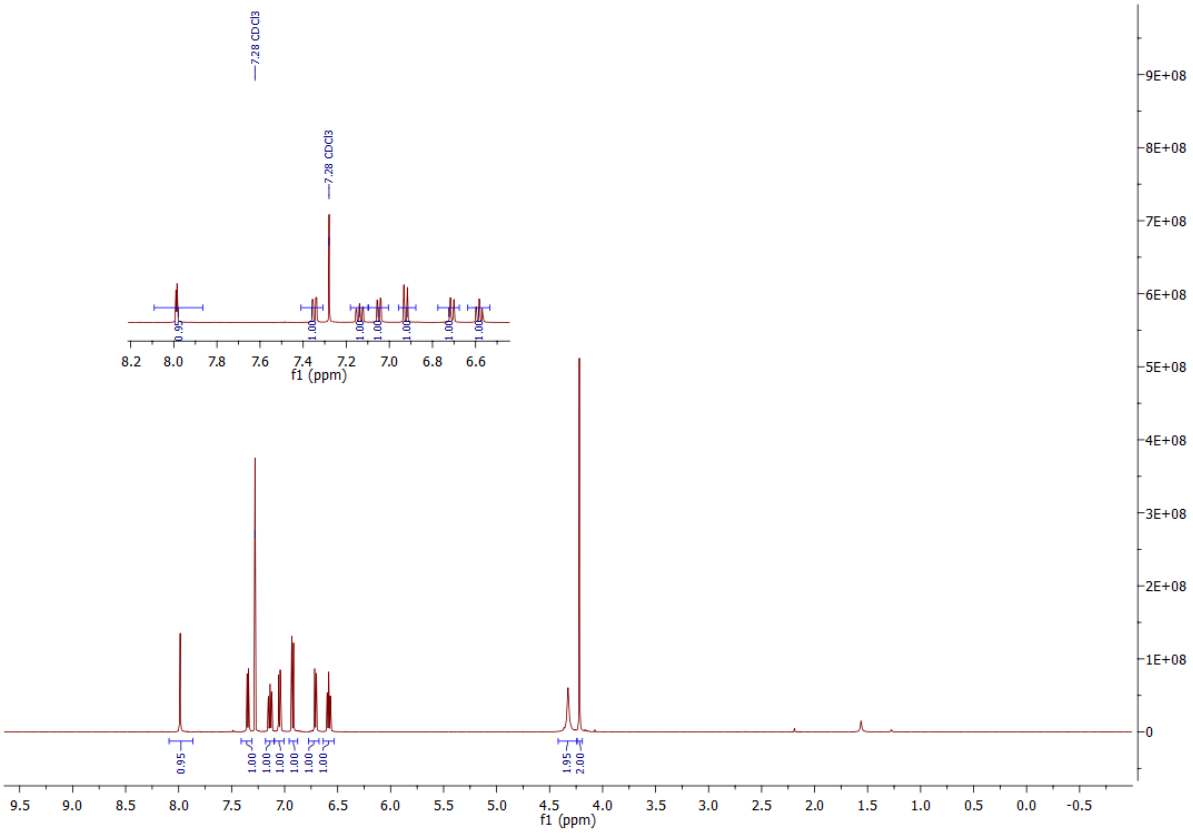

Figure S5: ^{1}H NMR spectrum of compound **13** measured in deuterated chloroform.

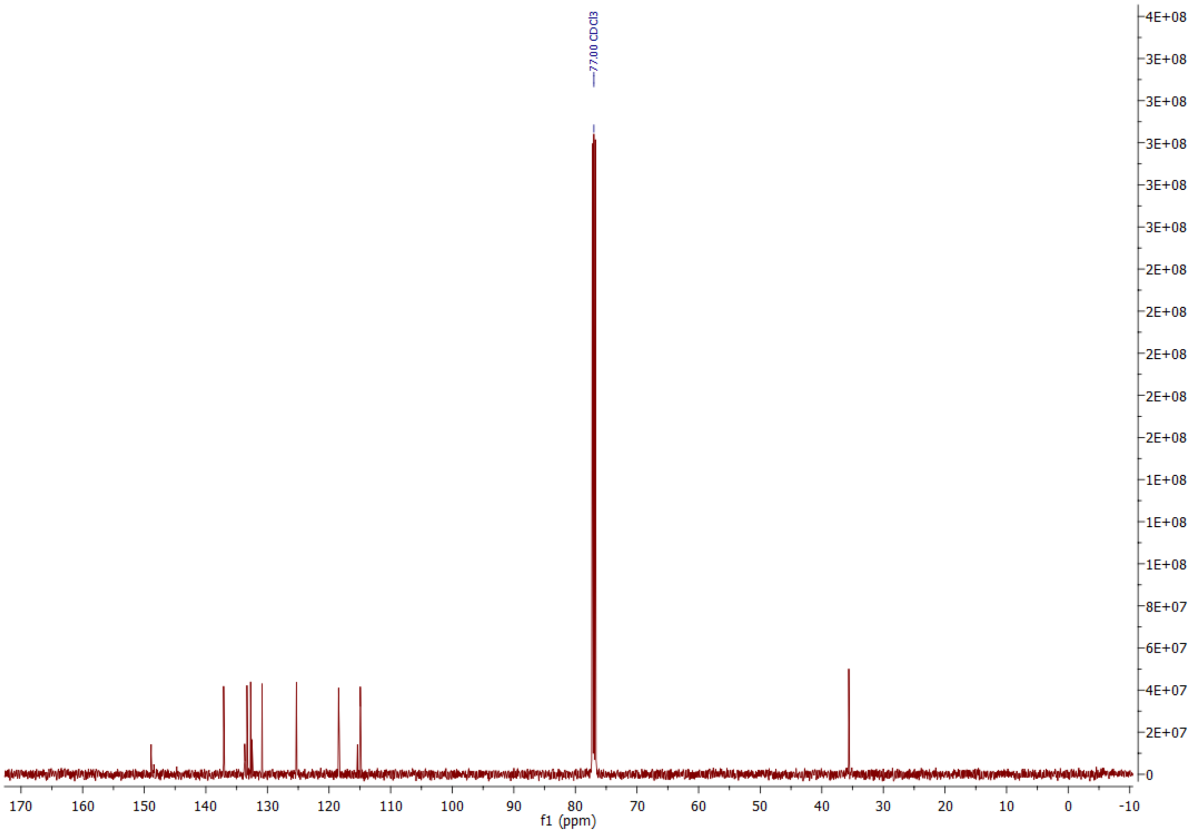

Figure S6: ^{13}C NMR spectrum of compound **13** measured in deuterated chloroform.

II.3.2. 2-((4-Bromo-2-nitrobenzyl)thio)aniline (14)

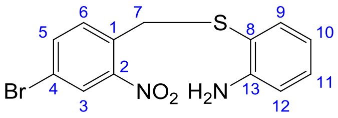

2,2'-Disulfanediyldianiline (**12**) (2.20 g, 8.86 mmol), sodium borohydride (919 mg, 24.2 mmol), 4-bromo-1-(bromomethyl)-2-nitrobenzene (**9**) (4.75 g, 16.1 mmol) in 50 mL dry THF.

Flash chromatography: cyclohexane/ethyl acetate, ethyl acetate: 6% → 50%.

An orange oil was obtained (4.62 g, 13.6 mmol, 84%).

R_f : 0.36 (cyclohexane/ethyl acetate, 3:1).

^{1}H NMR (600 MHz, acetone-d_6): δ = 8.13 (d, ^4J = 2.2 Hz, 1 H, *H*-3), 7.65 (dd, ^3J = 8.3 Hz, ^4J = 2.2 Hz, 1 H, *H*-5), 7.06 (m_c, 1 H, *H*-11), 7.05 (d, ^3J = 8.3 Hz, 1 H, *H*-6), 6.97 (dd, ^3J = 8.1 Hz, ^4J = 1.3 Hz, 1 H, *H*-12), 6.45 (dt, ^3J = 7.5 Hz, ^4J = 1.3 Hz, 1 H, *H*-10), 5.04 (bs, 1 H, NH_2), 4.24 (s, 2 H, *H*-7) ppm.

^{13}C NMR (150 MHz, acetone-d_6): δ = 151.0 (*C*-13), 150.0 (*C*-2), 137.5 (*C*-9), 136.4 (*C*-5), 134.8 (*C*-6), 134.0 (*C*-1), 131.5 (*C*-11), 128.5 (*C*-3), 121.2 (*C*-4), 117.9 (*C*-10), 115.5 (*C*-12), 115.0 (*C*-8), 35.6 (*C*-7) ppm.

IR (ATR): ṽ = 3475 (m), 3376 (m), 3107 (w), 1609 (s), 1561 (m), 1524 (vs), 1478 (s), 1450 (m), 1432 (m), 1337 (vs), 1306 (m), 1159 (w), 1216 (w), 1159 (m), 1112 (m), 1019 (w), 879 (m), 835 (m), 804 (m), 757 (vs), 734 (m), 699 (m), 638 (m), 523 (s), 356 (s), 418 (m), 406 (s) cm^{-1}.

MS (EI, 70 eV): m/z (%) = 337 (10) $[M]^+$, 213 (3) $[M-C_6H_6NS]^+$, 124 (100) $[M-C_7H_6BrNO_2]^+$.

MS (EI, HR, 70 eV): $[C_{13}H_{11}BrN_2O_2S]$, m/z = calc.: 337.9725, found: 337.9728.

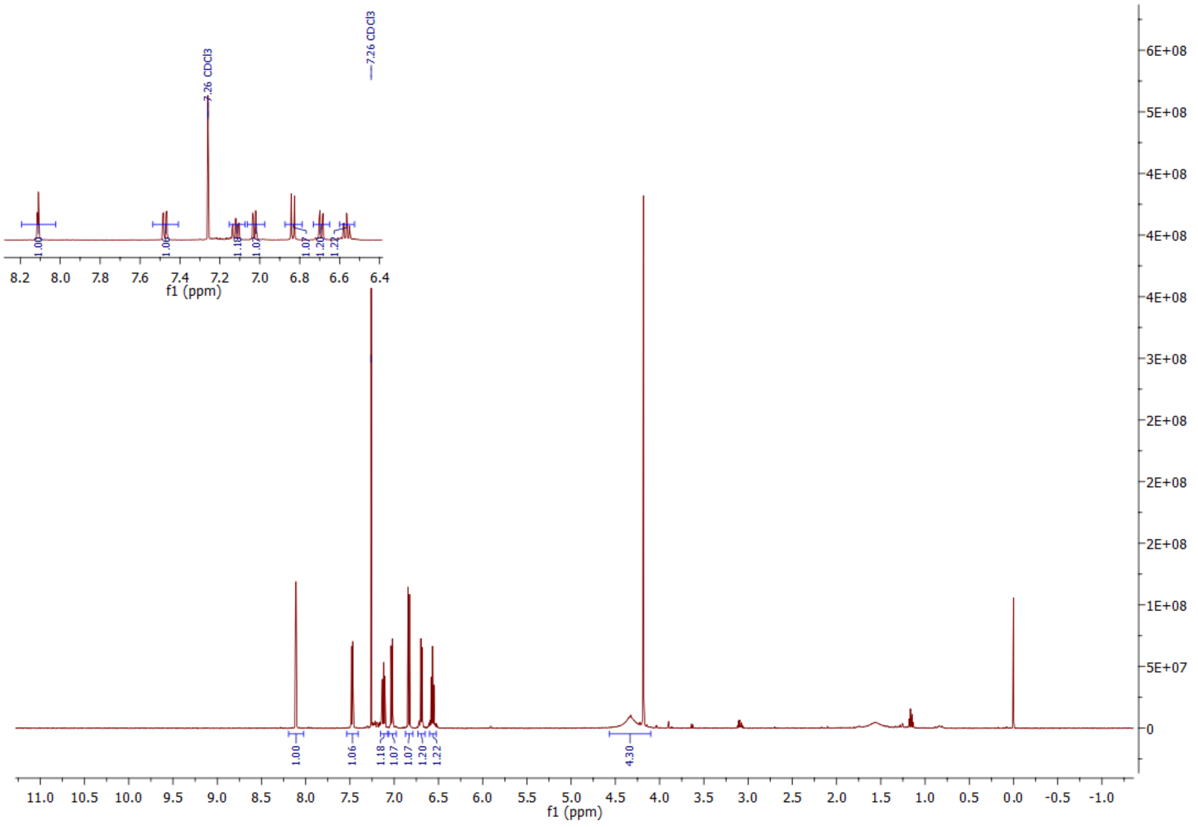

Figure S7: ^{1}H NMR spectrum of compound **14** measured in deuterated chloroform.

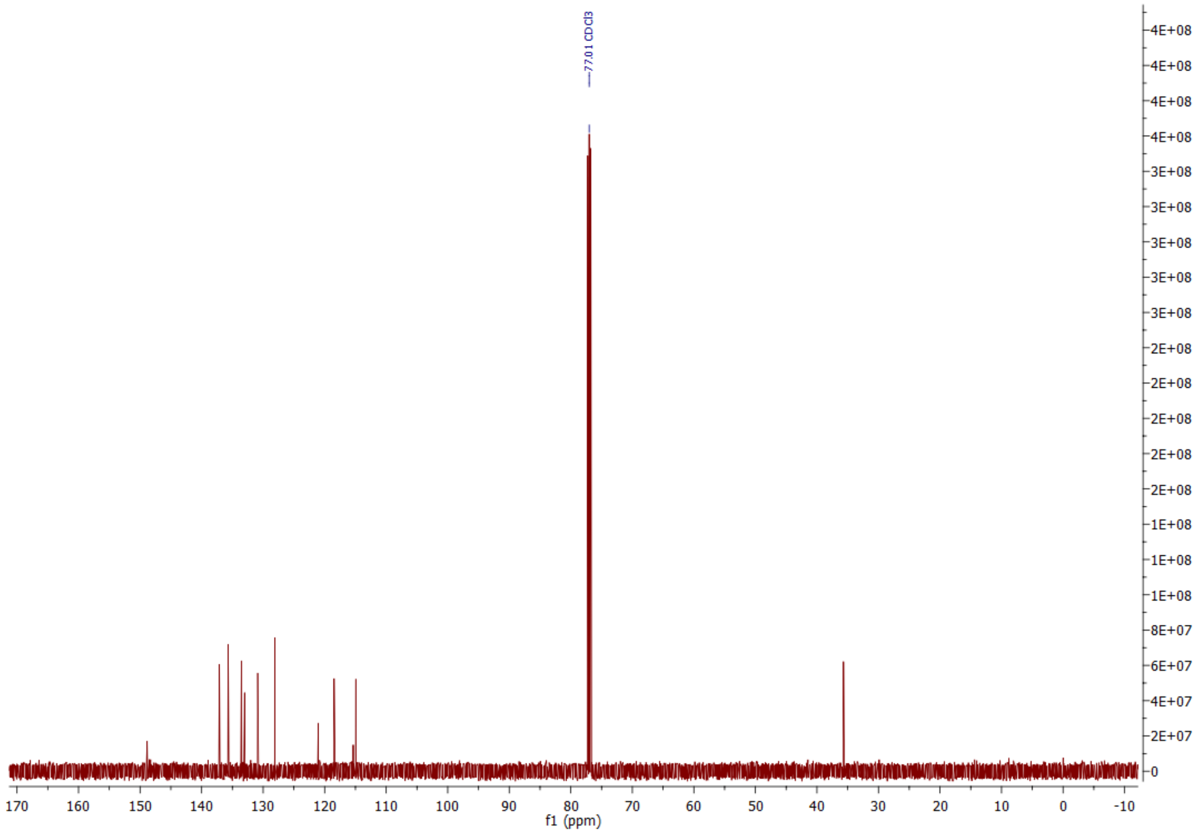

Figure S8: ^{13}C NMR spectrum of compound **14** measured in deuterated chloroform.

II.3.3. 2-((4-Iodo-2-nitrobenzyl)thio)aniline (15)

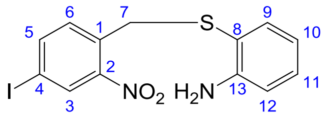

2,2'-Disulfanediyldianiline (**12**) (281 mg, 1.13 mmol), sodium borohydride (113 mg, 3.0 mmol), 4-iodo-1-(bromomethyl)-2-nitrobenzene (**10**) (707 mg, 2.0 mmol) in 15 mL dry THF.

Flash chromatography: cyclohexane/ethyl acetate, ethyl acetate: 7% → 60%.

An orange oil was obtained (506 mg, 1.31 mmol, 64%).

R_f : 0.5 (cyclohexane/ethyl acetate, 3:1).

^{1}H NMR (600 MHz, acetone-d_6): δ = 8.29 (d, ^{4}J = 1.9 Hz, 1 H, *H*-3), 7.84 (dd, ^{3}J = 8.1 Hz, ^{4}J = 1.9 Hz, 1 H, *H*-5), 7.06 (dt, ^{3}J = 7.7 Hz, ^{4}J = 1.6 Hz, 1 H, *H*-11), 6.97 (dd, ^{3}J = 7.7 Hz, ^{4}J = 1.5 Hz, 1 H, *H*-9), 6.90 (d, ^{3}J = 8.1 Hz, 1 H, *H*-6), 6.76 (dd, ^{3}J = 8.1 Hz, ^{4}J = 1.2 Hz, 1 H, *H*-12), 6.45 (dt, ^{3}J = 7.5 Hz, ^{4}J = 1.3 Hz, 1 H, *H*-10), 5.05 (bs, 2 H, NH_2), 4.22 (s, 2 H, *H*-7) ppm.

^{13}C NMR (150 MHz, acetone-d_6): δ = 151.0 (*C*-13), 149.9 (*C*-2), 142.3 (*C*-5), 137.5 (*C*-9), 134.8 (*C*-6), 134.5 (*C*-1), 134.2 (*C*-3), 131.5 (*C*-11), 117.9 (*C*-10), 115.5 (*C*-12), 115.1 (*C*-8), 91.9 (*C*-4), 35.7 (*C*-7) ppm.

IR (ATR): ῦ = 3460 (w), 3358 (w), 3062 (w), 1604 (m), 1556 (w), 1520 (vs), 1476 (s), 1447 (m), 1426 (m), 1339 (s), 1308 (m), 1259 (w), 1199 (w), 1158 (m), 1137 (m), 1075 (m), 1023 (w), 872 (m), 829 (m), 803 (m), 747 (s), 715 (m), 680 (m), 619 (m), 554 (m), 500 (m), 455 (m), 418 (m) cm^{-1}.

MS (EI, 70 eV): m/z (%) = 385 (16) $[M]^+$, 261 (3) $[M-C_6H_6NS]^+$, 124 (100) $[M-C_7H_6INO_2]^+$.

MS (EI, HR, 70 eV): $[C_{13}H_{11}IN_2O_2S]$, m/z = calc.: 385.9586, found: 385.9582.

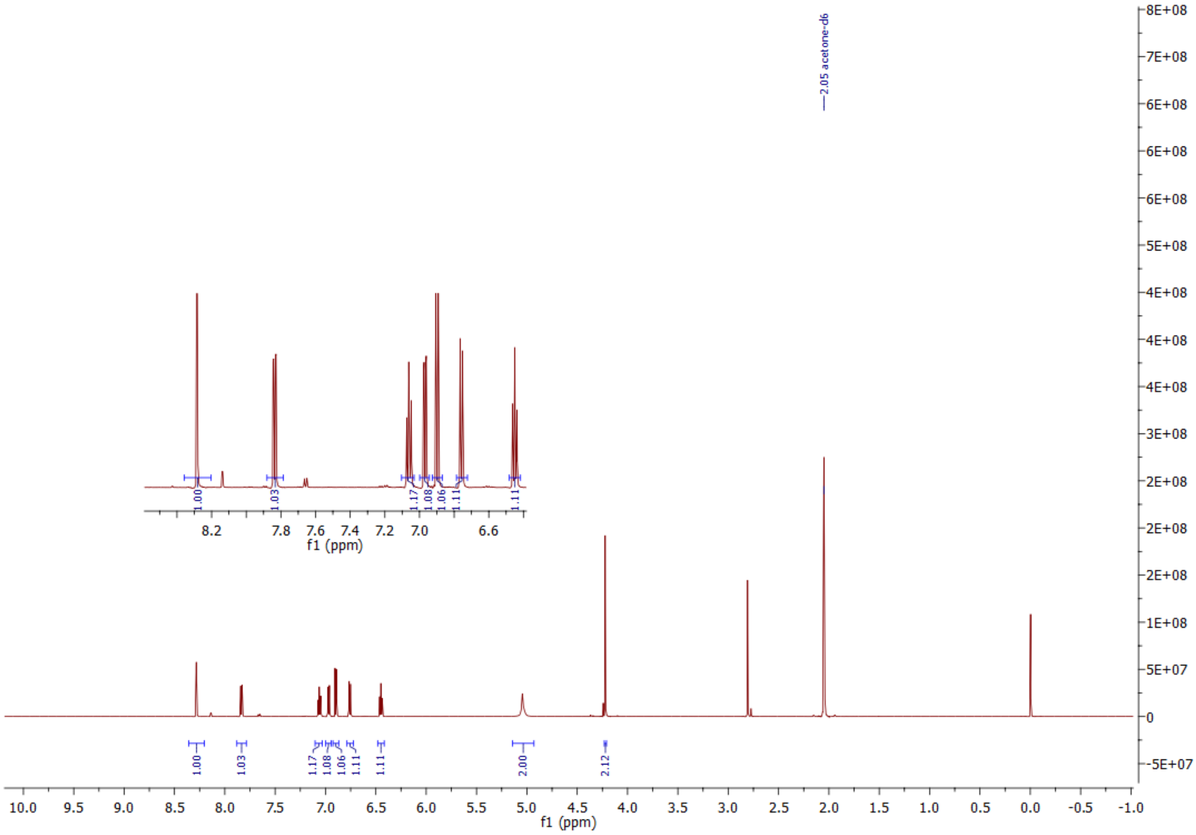

Figure S9: ^{1}H NMR spectrum of compound **15** measured in deuterated acetone.

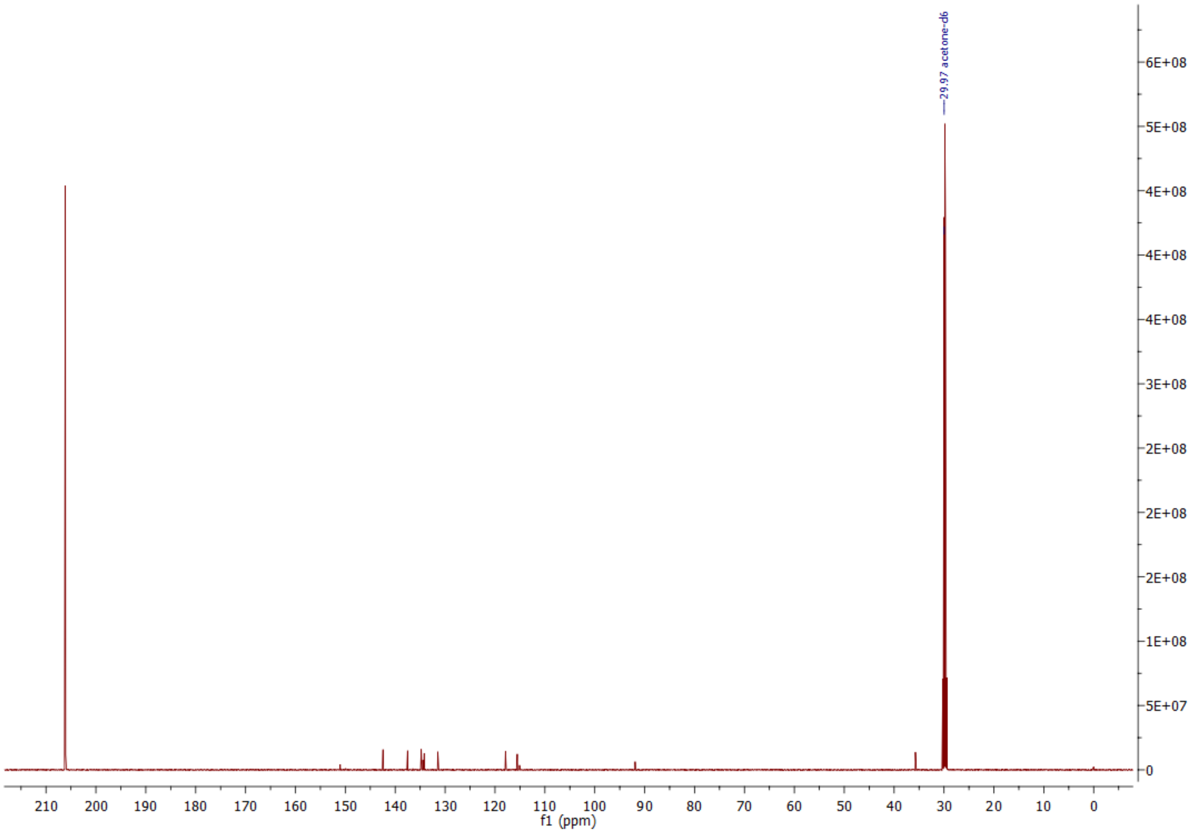

Figure S10: ^{13}C NMR spectrum of compound **15** measured in deuterated acetone.

II.3.4. 4-(((2-Aminophenyl)thio)methyl)-3-nitrobenzoic acid (16)

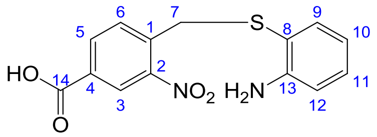

2,2’-Disulfanediyldianiline (**12**) (1.00 g, 4.03 mmol), sodium borohydride (416 mg, 11.0 mmol), 4-(bromomethyl)-3-nitrobenzoic acid (**11**) (1.91 g, 7.33 mmol) in 25 mL dry THF.

Flash chromatography: cyclohexane/ethyl acetate, ethyl acetate: 20% → 100%.

An orange solid was obtained (1.90 g, 6.24 mmol, 85%).

R*f* : 0.08 (cyclohexane/ethyl acetate, 1:1).

^{1}H NMR (600 MHz, DMSO-d$_6$): δ = 8.39 (d, ^3J = 1.59 Hz, 1 H, *H*-3), 7.99 (dd, ^3J = 8.04 Hz, ^4J = 1.59 Hz, 1 H, *H*-5), 7.25 (d, ^3J = 8.04 Hz, 1 H, *H*-6), 7.02 (m$_c$, 1 H, *H*-11), 6.85 (dd, ^3J = 7.74 Hz, ^4J = 1.45 Hz, 1 H, *H*-9), 6.68 (dd, ^3J = 8.10 Hz, ^4J = 0.98 Hz, 1 H, *H*-12), 6.36 (dt, ^3J = 6.43 Hz, ^4J = 1.08 Hz, 1 H, *H*-10), 5.35 (bs, 2 H, N*H*$_2$), 4.26 (s, 2 H, *H*-7) ppm.

^{13}C NMR (150 MHz, DMSO-d$_6$): δ = 165.3 (*C*-14), 150.0 (*C*-13), 148.0 (*C*-2), 137.9 (*C*-1), 136.0 (*C*-9), 133.1 (*C*-5), 132.8 (*C*-6), 130.9 (*C*-11), 130.33 (*C*-4), 125.5 (*C*-3), 116.2 (*C*-10), 114.4 (*C*-12), 113.1 (*C*-8), 34.6 (*C*-7) ppm.

IR (ATR): ῦ = 3477 (w), 3378 (w), 2358 (w), 1693 (s), 1608 (m), 1527 (m), 1479 (s), 1447 (m), 1344 (m), 1298 (s), 1148 (m), 917 (m), 849 (m), 821 (m), 754 (m), 730 (m), 699 (m), 639 (m), 501 (m), 426 (s), 408 (vs) cm^{-1}.

MS (EI, 70 eV): m/z (%) = 304 (15) [M]$^+$, 124 (100) [M-C$_8$H$_7$NO4]$^+$.

MS (EI, HR, 70 eV): [$C_{14}H_{12}N_2O_4S$], m/z = calc.: 304.0518, found: 304.0509.

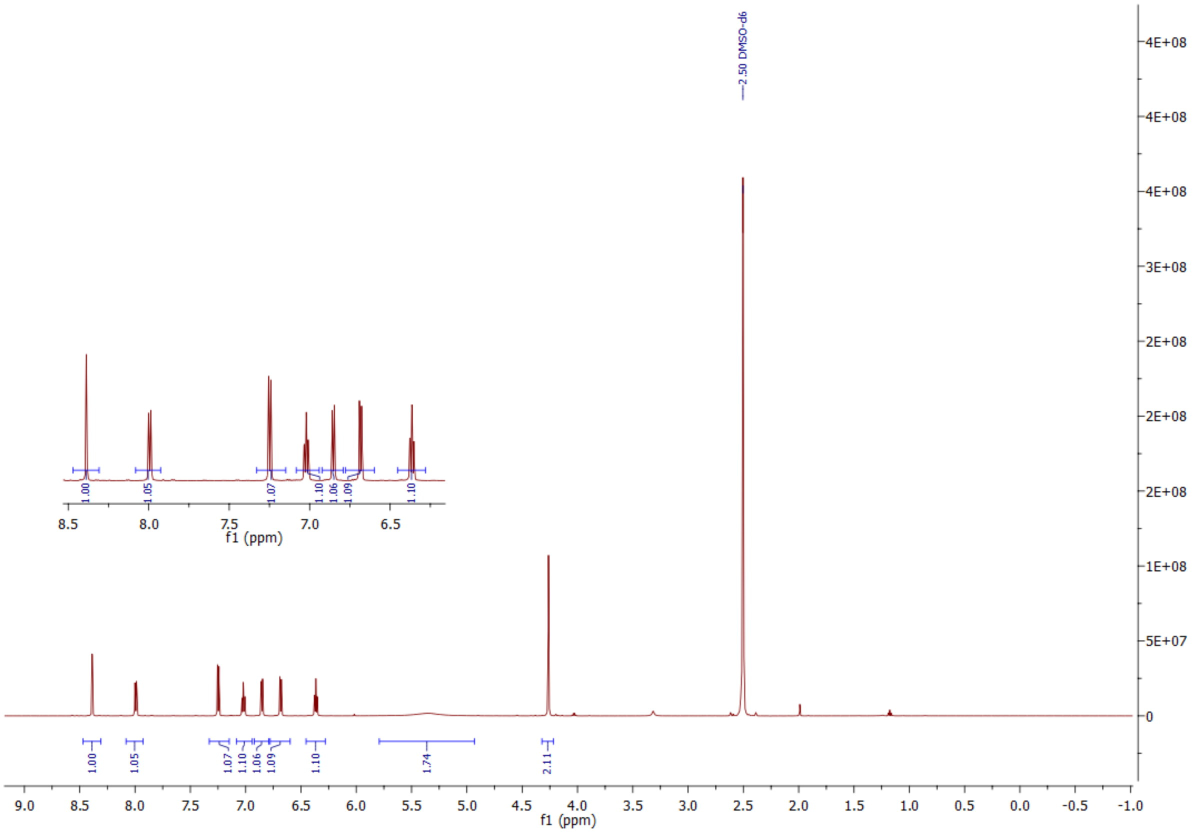

Figure S11: ^{1}H NMR spectrum of compound **16** measured in deuterated DMSO.

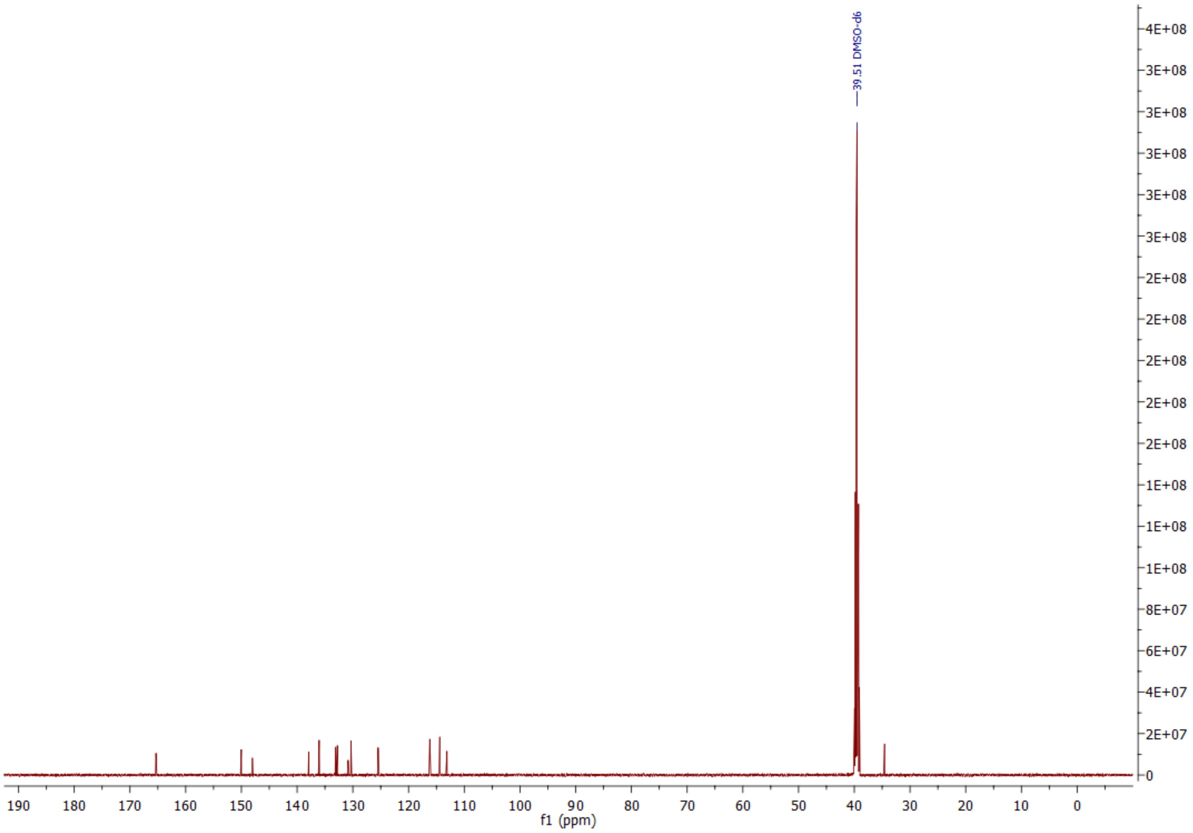

Figure S12: ^{13}C NMR spectrum of compound **16** measured in deuterated DMSO.

II.3.5. (4-(((2-Aminophenyl)thio)methyl)-3-nitrophenyl)methanol (17)

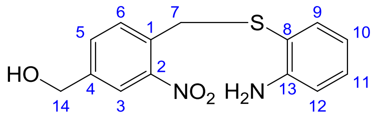

4-(((2-Aminophenyl)thio)methyl)-3-nitrobenzoic acid (**16**) (2.03 g, 6.68 mmol) was dissolved in 90 mL dry THF under nitrogen atmosphere. Borane tetrahydrofuran complex (19.3 mL, 1 M in THF) was added dropwise and the reaction was allowed to stir at room temperature for 22 h. 2 M hydrogen chloride solution was added and the organic layer was washed with saturated sodium bicarbonate solution (2 × 40 mL) and saturated sodium chloride solution (2 × 40 mL). The organic layer was dried over magnesium sulfate, filtered and the solvent was removed in vacuo. Due to the instability of the compound it was used without further purification in the next synthesis. It is not recommended to store the product **17**. The NMR shows small impurities. An orange solid was obtained (1.22 g, 4.16 mmol, 62%).

R_f : 0.11 (cyclohexane/ethyl acetate, 1:1).

^{1}H NMR (500 MHz, DMSO-d$_6$): δ = 7.91 (m$_c$, 1 H, *H*-3), 7.45 (m$_c$, 1 H, *H*-5), 7.13 (d, ^3J = 7.9 Hz, 1 H, *H*-6), 7.02 (m$_c$, 1 H, *H*-11), 6.89 (dd, ^3J = 7.7 Hz, ^4J = 1.6 Hz, 1 H, *H*-9), 6.69 (dd, ^3J = 8.1 Hz, ^4J = 1.3 Hz, 1 H, *H*-12), 6.38 (dt, ^3J = 7.4 Hz, ^4J = 1.3 Hz, 1 H, *H*-10), 5.46 (t, ^3J = 5.8 Hz, 1 H, O*H*), 5.34 (bs, 2 H, NH_2), 4.54 (d, ^3J = 5.8 Hz, 2 H, *H*-14), 4.19 (s, 2 *H*, *H*-7) ppm.

^{13}C NMR (125 MHz, DMSO-d$_6$): δ = 149.7 (*C*-13), 147.9 (*C*-2), 143.5 (*C*-4), 135.6 (*C*-9), 132.0 (*C*-6), 131.3 (*C*-1), 130.6 (*C*-5), 130.0 (*C*-11), 122.2 (*C*-3), 116.1 (*C*-10), 114.3 (*C*-12), 113.9 (*C*-8), 61.4 (*C*-14), 34.5 (*C*-7) ppm.

IR (ATR): ῦ = 3374 (w), 3314 (w), 2865 (w), 1608 (m), 1568 (w), 1522 (s), 1496 (m), 1475 (s), 1444 (m), 1327 (s), 1201 (w), 1151 (w), 1103 (w), 1045 (m), 935 (w), 891 (w), 832 (m), 815 (m), 747 (vs), 676 (m), 588 (m), 503 (m), 447 (m), 404 (m) cm^{-1}.

MS (EI, 70 eV): m/z (%) = 290 (12) $[M]^+$, 124 (100) $[M-C_8H_7NO4]^+$.

MS (EI, HR, 70 eV): $[C_{14}H_{14}N_2O_3S]$, m/z = calc.: 290.0725, found: 290.0733.

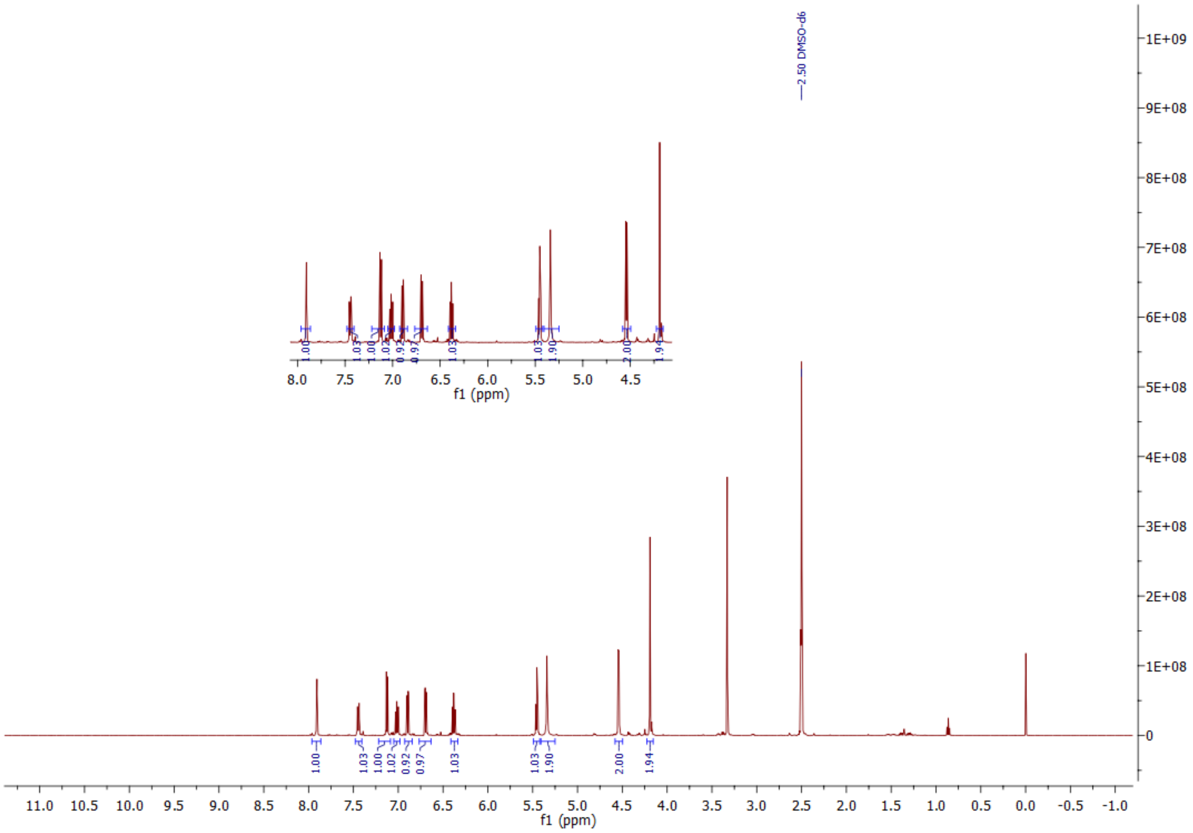

Figure S13: ^{1}H NMR spectrum of compound **17** measured in deuterated DMSO.

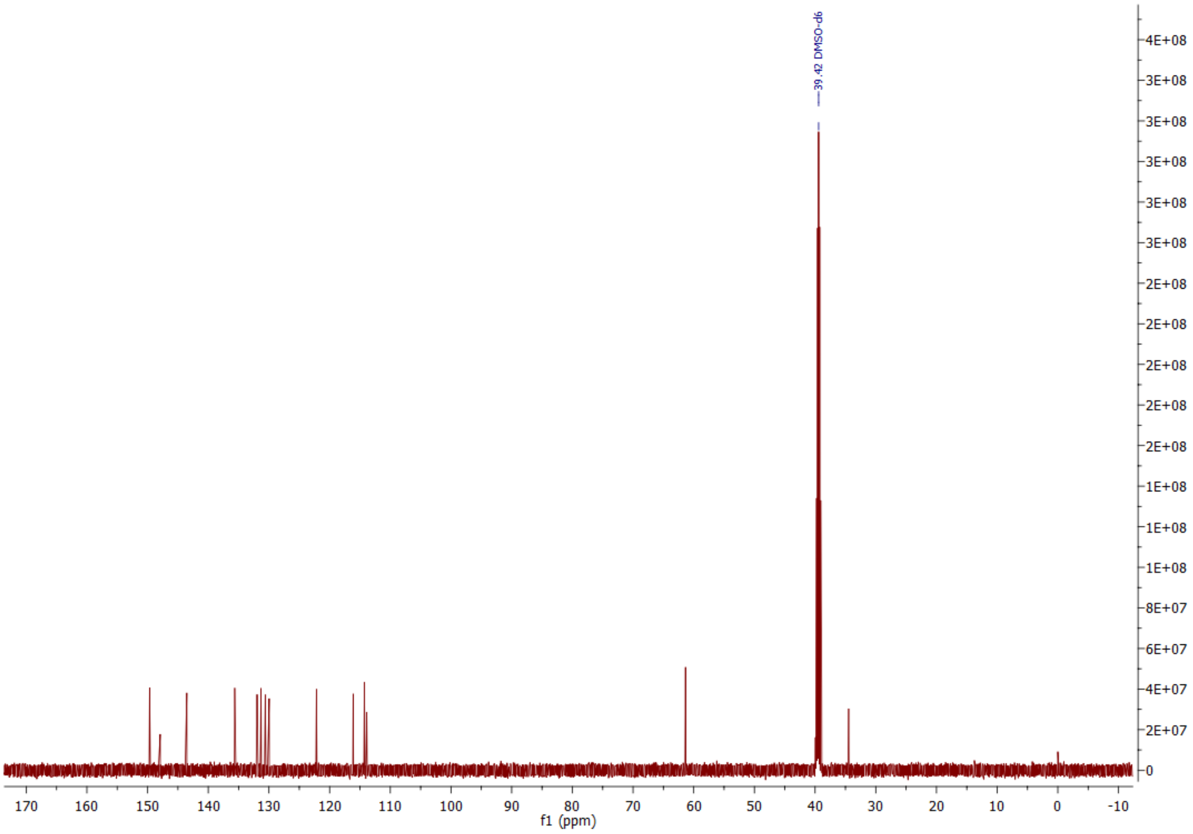

Figure S14: ^{13}C NMR spectrum of compound **17** measured in deuterated DMSO.

II.4. General method for the synthesis of the substituted S-diazocine derivatives 1–5:

The substituted thioether derivate **13**, **14**, **15**, **16** or **17** (1 equiv) was dissolved in ethanol (20 mL/mmol). Water (5 mL/mmol) and 2 M ammonia chloride solution (1.5 mL/mmol) were added and the reaction mixture was heated to 65 °C. Zinc powder (3.8 equiv) was added and the mixture was stirred at 65 °C for 10 min. The reaction mixture was filtered hot, the filtrate was diluted with ethanol (8 mL/mmol) and cooled with ice. The cooled solution was added dropwise to a 0 °C cooled iron(III) chloride hexahydrate (1.7 equiv) mixture in water (1.7 mL/mmol) and ice (1 g/mmol). The reaction mixture was stirred for 30 min at 0 °C, diluted with acetic acid (13 mL/mmol) and stirred at room temperature for 18 h. The reaction mixture was extracted with DCM, the combined organic layers were dried over magnesium sulfate, filtered and the solvent was evaporated in vacuo. The crude product was filtered over silica (ethyl acetate) and then purified by flash column chromatography.

II.4.1. (*Z*)-3-Chloro-12*H*-dibenzo[*b*,*f*][1,4,5]thiadiazocine (1)

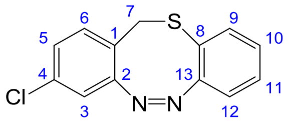

2-((4-Chloro-2-nitrobenzyl)thio)aniline (**13**) (1.46 g, 4.95 mmol), zinc powder (1.23 g, 18.8 mmol), iron(III)-chloride-hexahydrate (2.31 g, 8.58 mmol).

Flash chromatography: cyclohexane/ethyl acetate, ethyl acetate: 7% → 100%.

A yellow, crystalline solid was obtained (168 mg, 0.64 mmol, 13%).

melting point: 178 °C.

R*f*: 0.63 (cyclohexane/ethyl acetate, 5:1).

^{1}H NMR (500 MHz, acetone-d$_6$): δ = 7.30 (d, ^3J = 8.3 Hz, 1 H, *H*-6), 7.28 (dt, ^3J = 6.5 Hz, ^4J = 1.4 Hz, 1 H, *H*-10), 7.17 (dd, ^3J = 8.3 Hz, ^4J = 2.2 Hz, 1 H, *H*-9), 7.14 (dd, ^3J = 7.9 Hz, ^4J = 1.2 Hz, 1 H, *H*-5), 7.04 (dt, ^3J = 6.5 Hz, ^4J = 1.4 Hz, 1 H, *H*-11), 6.91 (d, ^4J = 2.2 Hz, 1 H, *H*-3), 6.82 (dd, ^3J = 6.5 Hz, ^4J = 1.4 Hz, 1 H, *H*-12), 3.97 (d, ^2J = 11.9 Hz, 1 H, *H*-7), 3.88 (d, ^2J = 11.9 Hz, 1 H, *H*-7) ppm.

^{13}C NMR (125 MHz, acetone-d$_6$): δ = 159.2 (*C*-8), 158.6 (*C*-4), 134.4 (*C*-1), 134.2 (*C*-9), 132.0 (*C*-6), 129.1 (*C*-10), 128.3 (*C*-5), 128.2 (*C*-11), 124.3 (*C*-2), 122.6 (*C*-13), 120.2 (*C*-12), 118.0 (*C*-3), 24.5 (*C*-7) ppm.

IR (ATR): ύ = 3049 (w), 2253 (w), 1591 (m), 1458 (m), 1243 (w), 1162 (w), 1104 (m), 953 (w), 923 (w), 896 (m), 879 (m), 864 (m), 847 (m), 826 (s), 755 (vs), 731 (vs), 702 (m), 667 (m), 650 (m), 603 (m), 581 (w), 526 (m) cm^{-1}.

MS (EI, 70 eV): m/z = 260 (100) [M]$^+$.

MS (EI, HR, 70 eV): $C_{13}H_9ClN_2S$, m/z =calc.: 260.0175, found: 260.0171.

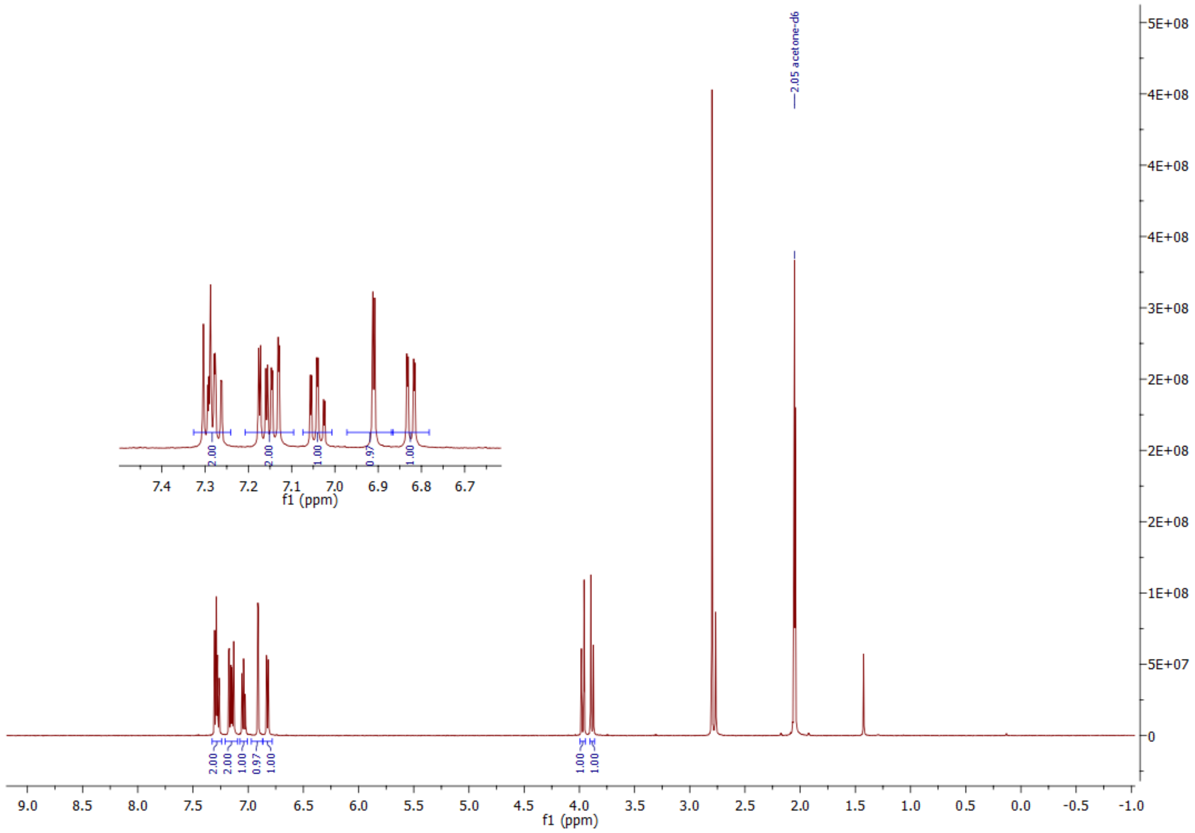

Figure S15: ^{1}H NMR spectrum of compound **1** measured in deuterated acetone.

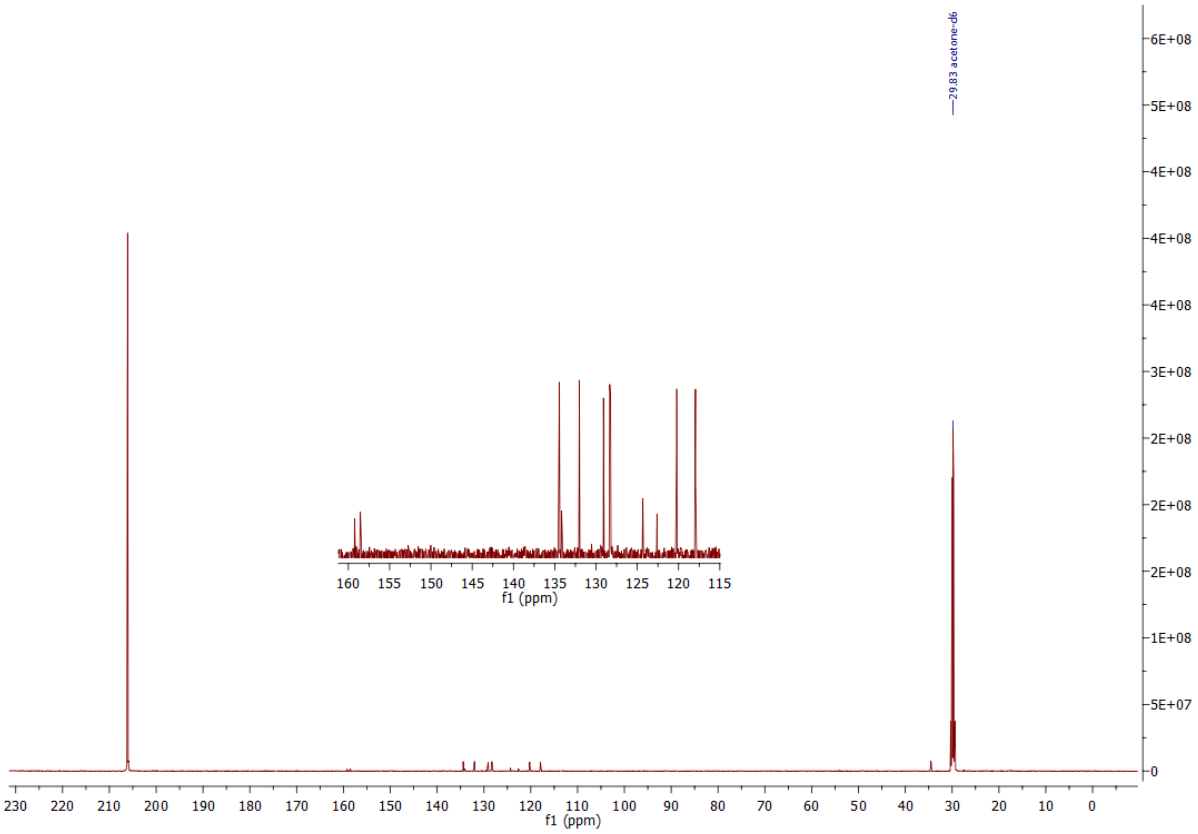

Figure S16: ^{13}C NMR spectrum of compound **1** measured in deuterated acetone.

II.4.2. (*Z*)-3-Bromo-12*H*-dibenzo[*b,f*][1,4,5]thiadiazocine (2)

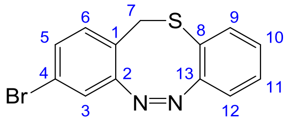

2-((4-Bromo-2-nitrobenzyl)thio)aniline (**14**) (3.93 g, 9.99 mmol), zinc powder (2.48 g, 38.0 mmol), iron(III) chloride hexahydrate (5.40 g, 20.0 mmol).

Flash chromatography: cyclohexane/ethyl acetate, ethyl acetate: 5% → 50%.

A yellow, crystalline solid was obtained (520 mg, 1.68 mmol, 17%).

melting point: 174 °C.

R_f: 0.61 (cyclohexane/ethyl acetate, 5:1).

^{1}H NMR (500 MHz, $CDCl_3$): δ = 7.20 (dt, ^3J = 7.8 Hz, ^4J = 1.3 Hz, 1 H, *H*-10), 7.12 (dd, ^3J = 7.8 Hz, ^4J = 1.3 Hz, 1 H, *H*-12), 7.08-7.04 (m, 2 H, *H*-5, *H*-3), 6.98 (dt, ^3J = 7.7 Hz, ^4J = 1.4 Hz, 1 H, *H*-11), 6.74-6.70 (m, 2 H, *H*-6, *H*-9), 4.05 (d, ^2J = 11.8 Hz, 1 H, *H*-7), 3.64 (d, ^2J = 11.8 Hz, 1 H, *H*-7) ppm.

^{13}C NMR (125 MHz, $CDCl_3$): δ = 157.9 (*C*-2), 157.4 (*C*-8), 133.8 (*C*-1), 133.7 (*C*-12), 130.5 (*C*-5), 128.1 (*C*-10), 127.6 (*C*-3), 127.3 (*C*-11), 122.9 (*C*-13), 121.7 (*C*-4), 119.2 (*C*-6), 117.2 (*C*-9), 34.3 (*C*-7) ppm.

IR (ATR): ύ = 3050 (w), 2359 (w), 1586 (m), 1562 (m), 1471 (m), 1458 (m), 1391 (m), 1242 (w), 1160 (w), 1099 (m), 1074 (m), 1031 (w), 952 (w), 922 (w), 896 (m), 841 (m), 823 (s), 754 (s), 731 (vs), 699 (m), 661 (m), 644 (m), 599 (m), 496 (s), 463 (s), 426 (s), 408 (vs) cm^{-1}.

MS (EI, 70 eV): m/z = 303 (100) $[M]^+$, 225 (36) $[M-Br]^+$.

MS (EI, HR, 70 eV): $C_{13}H_9BrN_2S$, m/z =calc.: 303.9670, found: 303.9665.

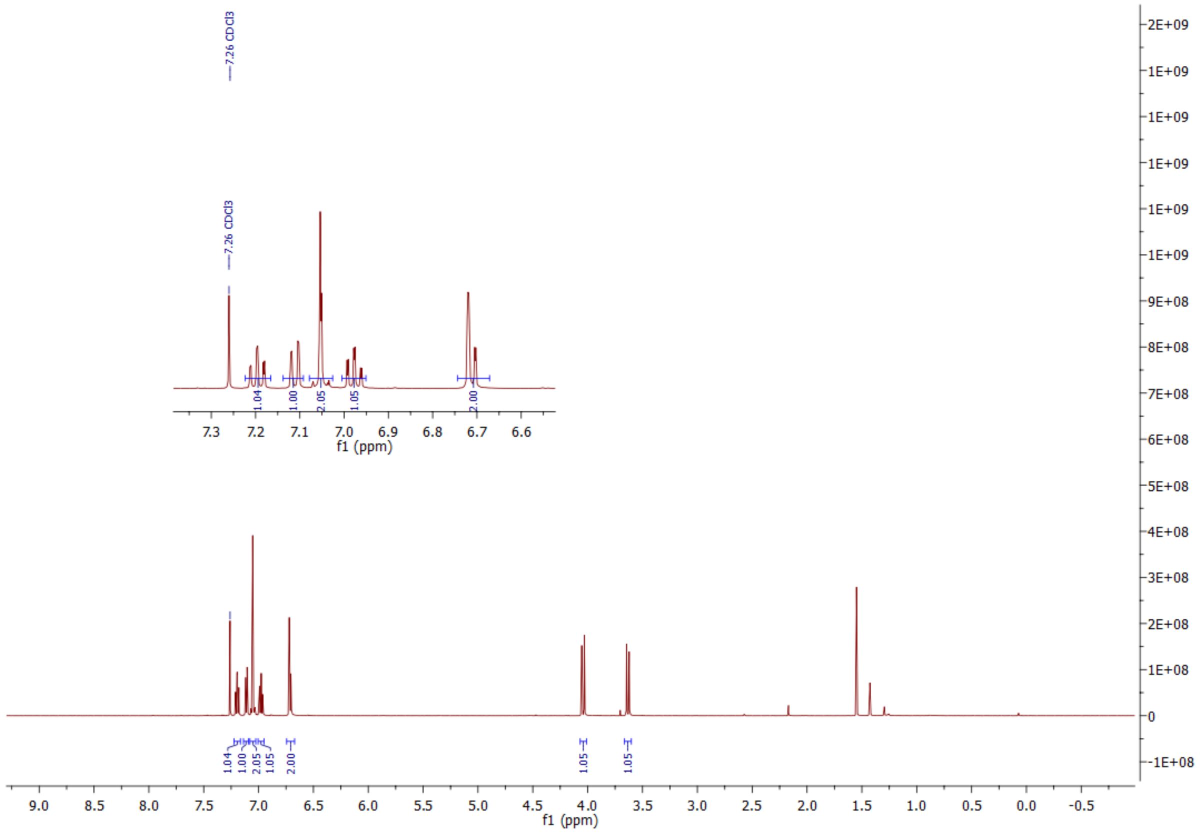

Figure S17: ^{1}H NMR spectrum of compound **2** measured in deuterated chloroform.

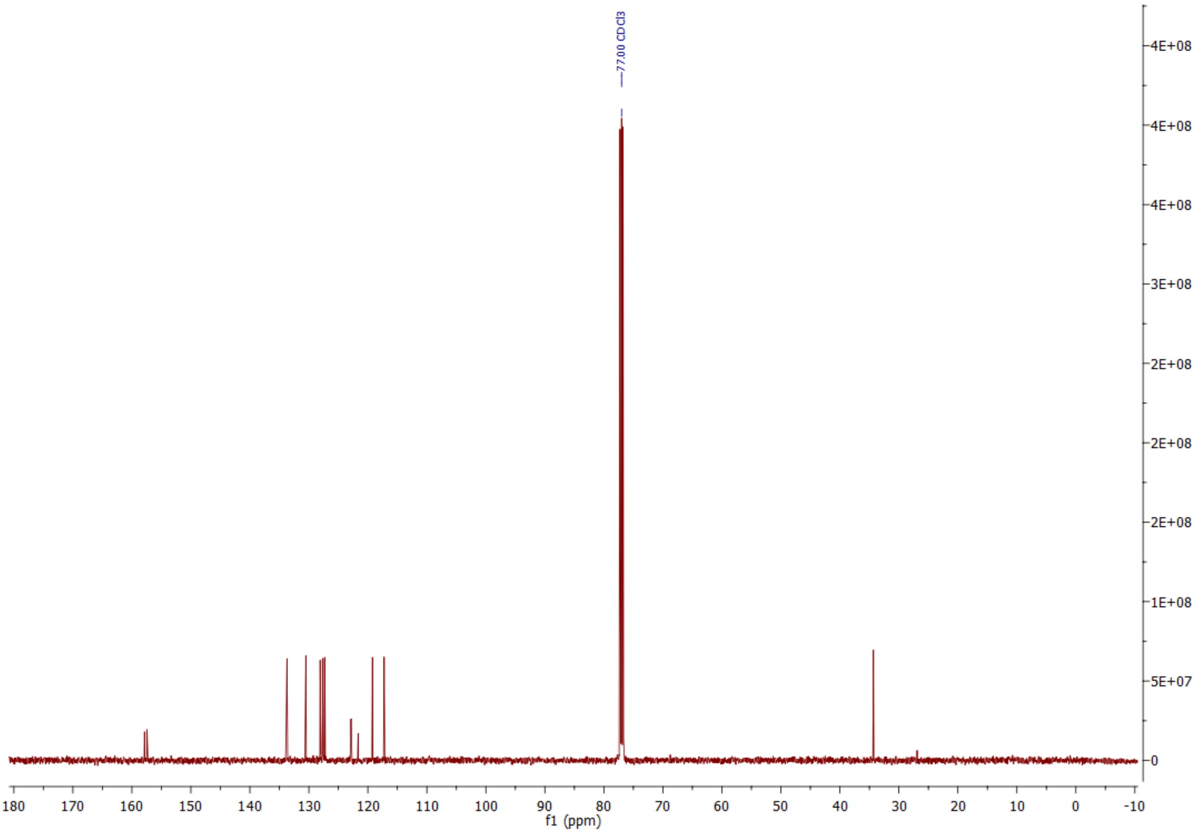

Figure S18: ^{13}C NMR spectrum of compound **2** measured in deuterated chloroform.

II.4.3. (*Z*)-3-Iodo-12*H*-dibenzo[*b*,*f*][1,4,5]thiadiazocine (3)

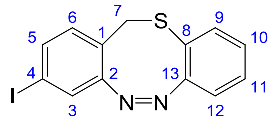

2-((4-Iodo-2-nitrobenzyl)thio)aniline (**15**) (759 mg, 2.00 mmol), zinc powder (488 mg, 7.46 mmol), iron(III)-chloride-hexahydrate (903 mg, 3.34 mmol).

Flash chromatography: cyclohexane/ethyl acetate, ethyl acetate: 7% → 60%.

A yellow, crystalline solid was obtained (187 mg, 0.52 mmol, 27%).

melting point: 157 °C.

R_f: 0.57 (cyclohexane/ethyl acetate, 3:1).

^{1}H NMR (600 MHz, acetone-d_6): δ = 7.52 (dd, ^3J = 8.1 Hz, ^4J = 1.7 Hz, 1 H, *H*-5), 7.28 (dt, ^3J = 8.0 Hz, ^4J = 1.3 Hz, 1 H, *H*-10), 7.21 (d, ^4J = 1.7 Hz, 1 H, *H*-3), 7.14 (dd, ^3J = 7.9 Hz, ^4J = 1.0 Hz, 1 H, *H*-12), 7.09 (d, ^3J = 8.1 Hz, 1 H, *H*-6), 7.64 (dt, ^3J = 6.4 Hz, ^4J = 1.4 Hz, 1 H, *H*-11), 6.83 (dd, ^3J = 8.0 Hz, ^4J = 1.3 Hz, 1 H, *H*-9), 3.95 (d, ^2J = 11.8 Hz, 1 H, *H*-7), 3.86 (d, ^2J = 11.8 Hz, 1 H, *H*-7) ppm.

^{13}C NMR (150 MHz, acetone-d_6): δ = 159.4 (*C*-2), 158.6 (*C*-8), 137.4 (*C*-5), 134.4 (*C*-12), 132.2 (*C*-6), 129.1 (*C*-10), 128.3 (*C*-11), 126.5 (*C*-3), 125.3 (*C*-1), 122.6 (*C*-13), 120.3 (*C*-9), 93.2 (*C*-4), 34.7 (*C*-7) ppm.

IR (ATR): ύ = 3041 (w), 2252 (w), 2179 (w), 1578 (m), 1457 (m); 1378 (m), 1095 (m), 1064 (m), 864 (m), 836 (m), 820 vs), 756 (s), 733 (s), 696 (m), 642 (m), 663 (w), 574 (w), 521 (w), 498 (m), 460 (s), 443 (m) cm^{-1}.

MS (EI, 70 eV): m/z = 351 (100) $[M]^+$, 225 (55) $[M-I]^+$.

MS (EI, HR, 70 eV): $C_{13}H_9IN_2S$, m/z =calc.: 351.9531, found: 351.9526.

For crystallographic data of compound **3** see chapter V. (page 31).

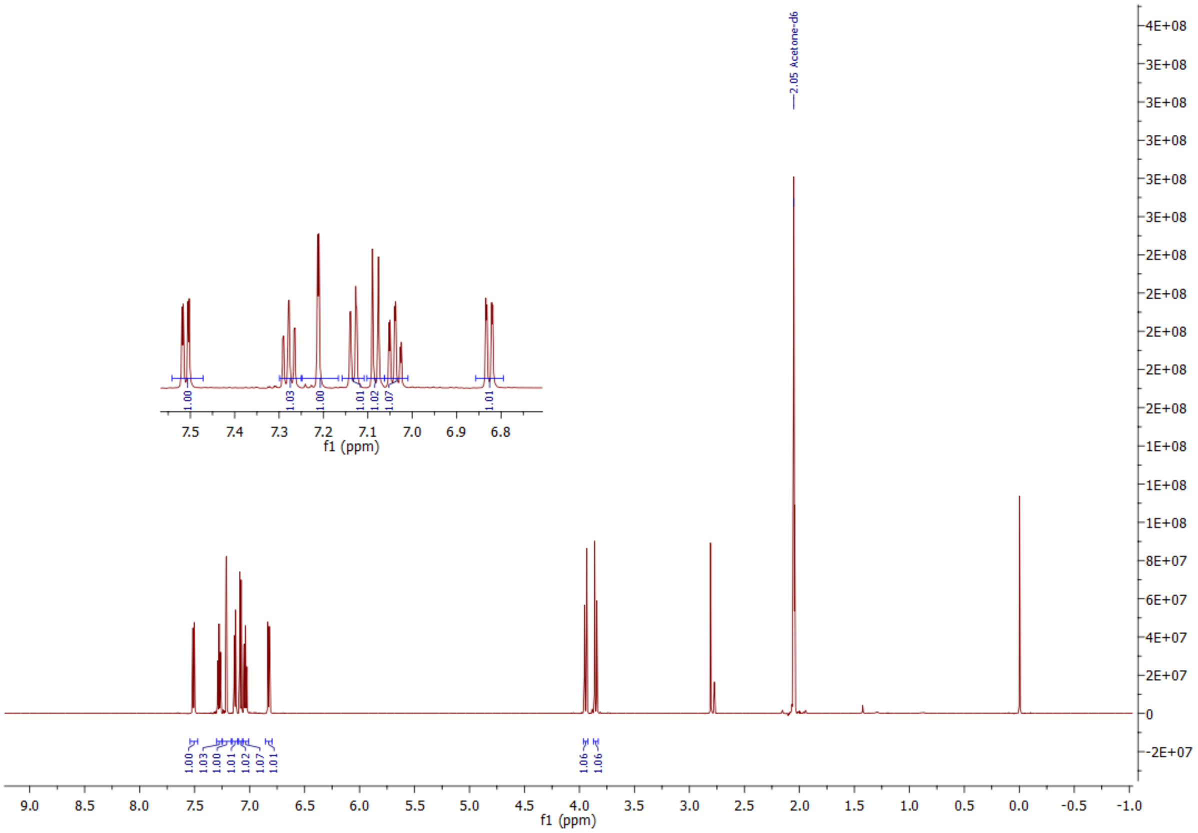

Figure S19: ^{1}H NMR spectrum of compound **3** measured in deuterated acetone.

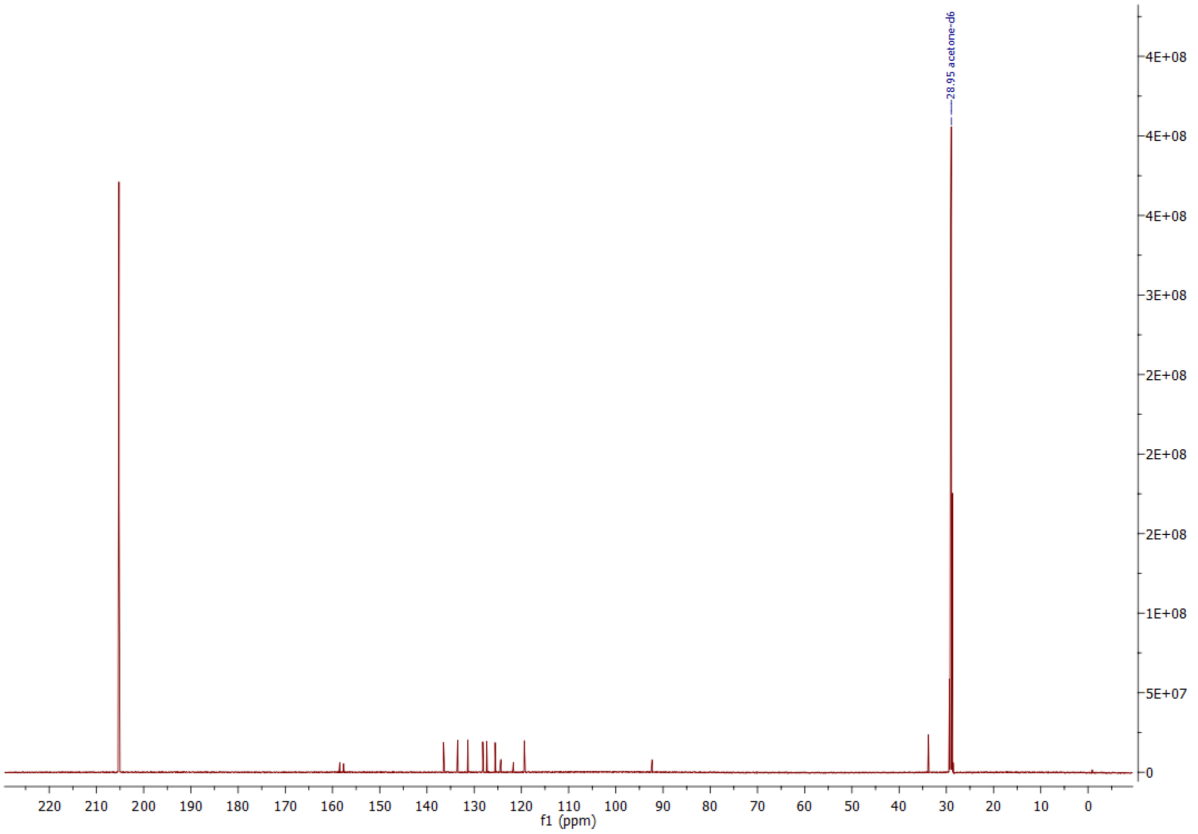

Figure S20: ^{13}C NMR spectrum of compound **3** measured in deuterated acetone.

II.4.4. (*Z*)-12*H*-Dibenzo[*b*,*f*][1,4,5]thiadiazocine-3-carboxylic acid (4)

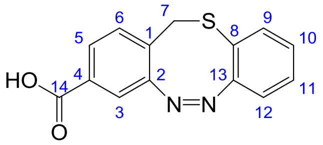

4-(((2-Aminophenyl)thio)methyl)-3-nitrobenzoic acid (**16**) (2.20 g, 7.23 mmol), zinc powder (1.79 g, 27.4 mmol), iron(III)-chloride-hexahydrate (3.86 g, 14.3 mmol).

Flash chromatography: cyclohexane/ethyl acetate, ethyl acetate: 7% → 100%.

A yellow, crystalline solid was obtained (383mg, 1.42 mmol, 20%).

melting point: 220 °C.

R*f*: 0.38 (cyclohexane/ethyl acetate, 3:1).

^{1}H NMR (600 MHz, acetone-d$_6$): δ = 7.81 (dd, ^{3}J = 8.0 Hz, ^{4}J = 1.7 Hz, 1 H, *H*-5), 7.44 (d, ^{3}J = 8.0 Hz, 1 H, *H*-6), 7.41 (d, ^{4}J = 1.7 Hz, 1 H, *H*-3), 7.27 (dt, ^{3}J = 7.8 Hz, ^{4}J = 1.3 Hz, 1 H, *H*-11), 7.14 (dd, ^{3}J = 7.8 Hz, ^{4}J = 1.2 Hz, 1 H, *H*-9), 7.02 (dt, ^{3}J = 7.8 Hz, ^{4}J = 1.3 Hz, 1 H, *H*-10), 6.88 (dd, ^{3}J = 7.8 Hz, ^{4}J = 1.3 Hz, 1 H, *H*-12), 4.01 (d, ^{2}J = 11.6 Hz, 1 H, *H*-7), 3.98 (d, ^{2}J = 11.6 Hz, 1 H, *H*-7) ppm.

^{13}C NMR (150 MHz, acetone-d$_6$): δ = 165.5 (*C*-14), 157.7 (*C*-13), 157.4 (*C*-2), 133.4 (*C*-9), 130.7 (*C*-4), 129.8 (*C*-6), 129.5 (*C*-1), 128.6 (*C*-5), 128.3 (*C*-11), 127.3 (*C*-10), 121.5 (*C*-8), 119.4 (*C*-12), 118.2 (*C*-3), 34.0 (*C*-7) ppm.

IR (ATR): ṽ = 1688 (s), 1609 (w), 1570 (w), 1429 (m), 1305 (s), 1270 (m), 1237 (w), 1210 (w), 1084 (w), 1065 (w), 1034 (w), 944 (m), 913 (w), 877 (w), 847 (m), 794 (w), 757 (vs), 727 (m), 697 (m), 647 (w), 599 (w), 552 (m), 505 (m), 457 (w), 439 (w) cm^{-1}.

MS (EI, 70 eV): m/z = 270 (100) $[M]^+$, 225 (26) $[M-CHO_2]^+$.

.

MS (EI, HR, 70 eV): $C_{14}H_{10}N_2O_2S$, m/z =calc.: 270.0463, found: 270.0460.

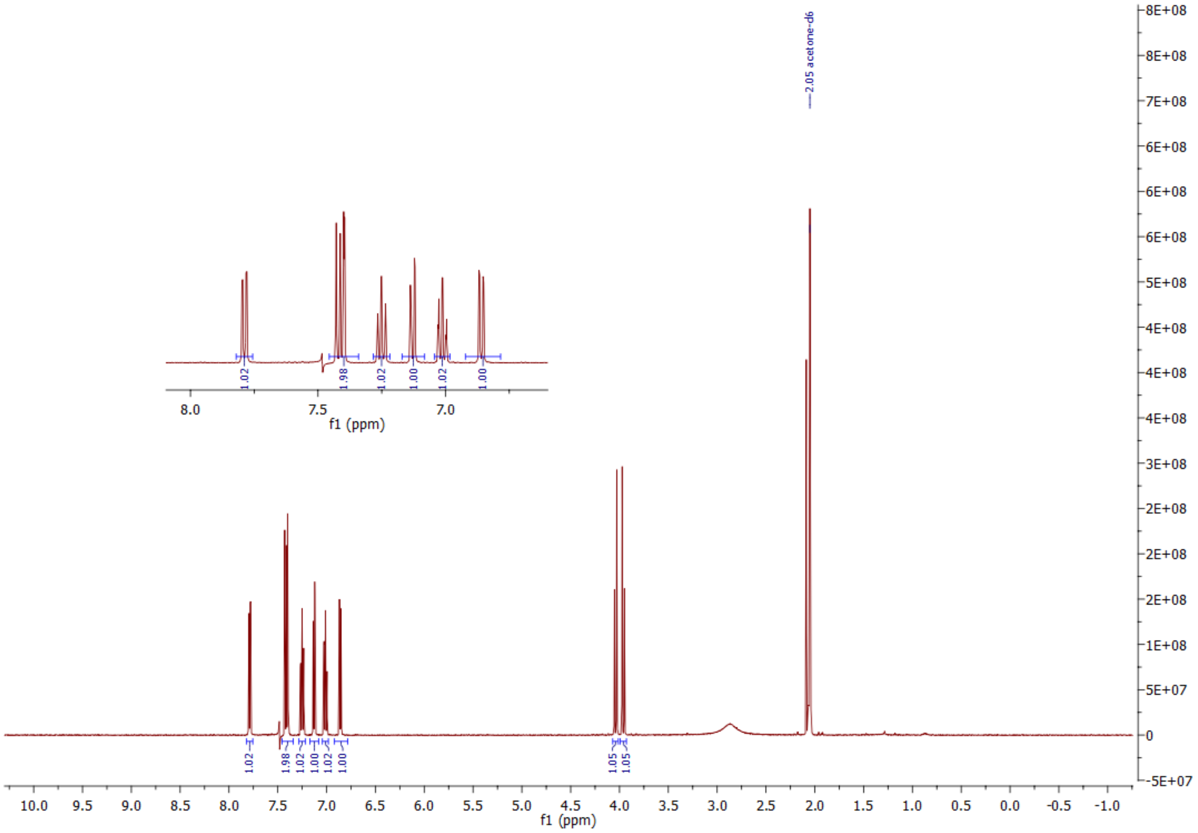

Figure S21: ^{1}H NMR spectrum of compound **4** measured in deuterated acetone.

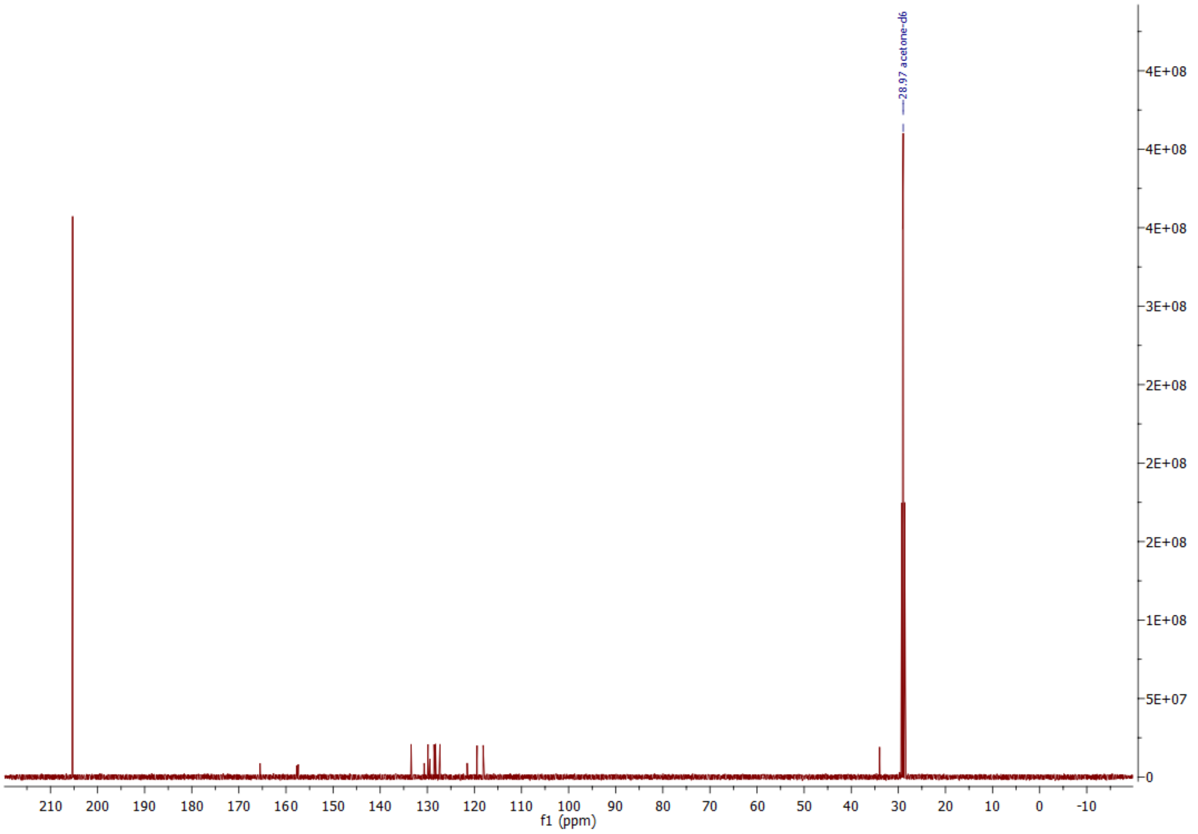

Figure S22: ^{13}C NMR spectrum of compound **4** measured in deuterated acetone.

II.4.5. (*Z*)-(12*H*-Dibenzo[*b,f*][1,4,5]thiadiazocin-3-yl)methanol (5)

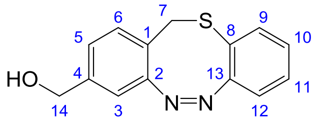

(4-(((2-Aminophenyl)thio)methyl)-3-nitrophenyl)methanol (**17**) (1.20 g, 4.14 mmol), zinc powder (1.03 g, 15.8 mmol), iron(III)-chloride-hexahydrate (2.21 g, 8.16 mmol).

Flash chromatography: cyclohexane/ethyl acetate, ethyl acetate: 12% → 100% and reversed flash chromatography: H_2O/acetonitrile, acetonitrile: 8% → 66%.

A yellow oil was obtained (60 mg, 234 µmol, 6%).

R$_f$: 0.44 (cyclohexane/ethyl acetate, 1:1).

R$_f$: 0.57 (RP, H_2O/acetonitrile, 1:2).

^{1}H NMR (500 MHz, acetone-d$_6$): δ = 7.25-7.18 (m, 2 H, *H*-6, *H*-11), 7.13-7.08 (m, 2 H, *H*-5, *H*-12), 7.00 (dt, ^3J = 7.6 Hz, ^4J = 1.4 Hz, 1 H, *H*-10), 6.80 (s, 1 H, *H*-3), 6.76 (dd, ^3J = 7.9 Hz, ^4J = 1.3 Hz, 1 H, *H*-9), 4.53 (bs, 2 H, *H*-14), 4.28 (bs, 1 H, O*H*), 3.97 (d, ^2J = 11.8 Hz, 1 H, *H*-7), 3.83 (d, ^2J = 11.8 Hz, 1 H, *H*-7) ppm.

^{13}C NMR (125 MHz, acetone-d$_6$): δ = 158.7 (*C*-13), 158.5 (*C*-2), 144.4 (*C*-1), 134.1 (*C*-12), 130.1 (*C*-6), 128.7 (*C*-11), 127.9 (*C*-10), 126.1 (*C*-5), 123.1 (*C*-4), 123.0 (*C*-8), 120.3 (*C*-9), 115.5 (*C*-3), 63.7 (*C*-14), 34.9 (*C*-7) ppm.

IR (ATR): ύ = 3362 (w), 3053 (w), 2931 (w), 2867 (w), 1708 (w), 1609 (w), 1565 (w), 1525 (w), 1491 (w), 1456 (m), 1428 (m), 1413 (m), 1358 (w), 1241 (w), 1200 (w), 1152 (w), 1124 (w), 1091 (w), 1031 (m), 904 (w), 873 (w), 828 (m), 789 (w), 758 (vs), 735 (vs), 692 (m), 673 (m); 656 (m), 630 (m), 550 (m), 528 (m), 495 (m), 463 (m), 444 (m), 406 (m) cm^{-1}.

MS (EI, 70 eV): m/z = 256 (50) [M]$^+$.

.

MS (EI, HR, 70 eV): $C_{14}H_{12}N_2OS$, m/z =calc.: 256.0670, found: 256.0666.

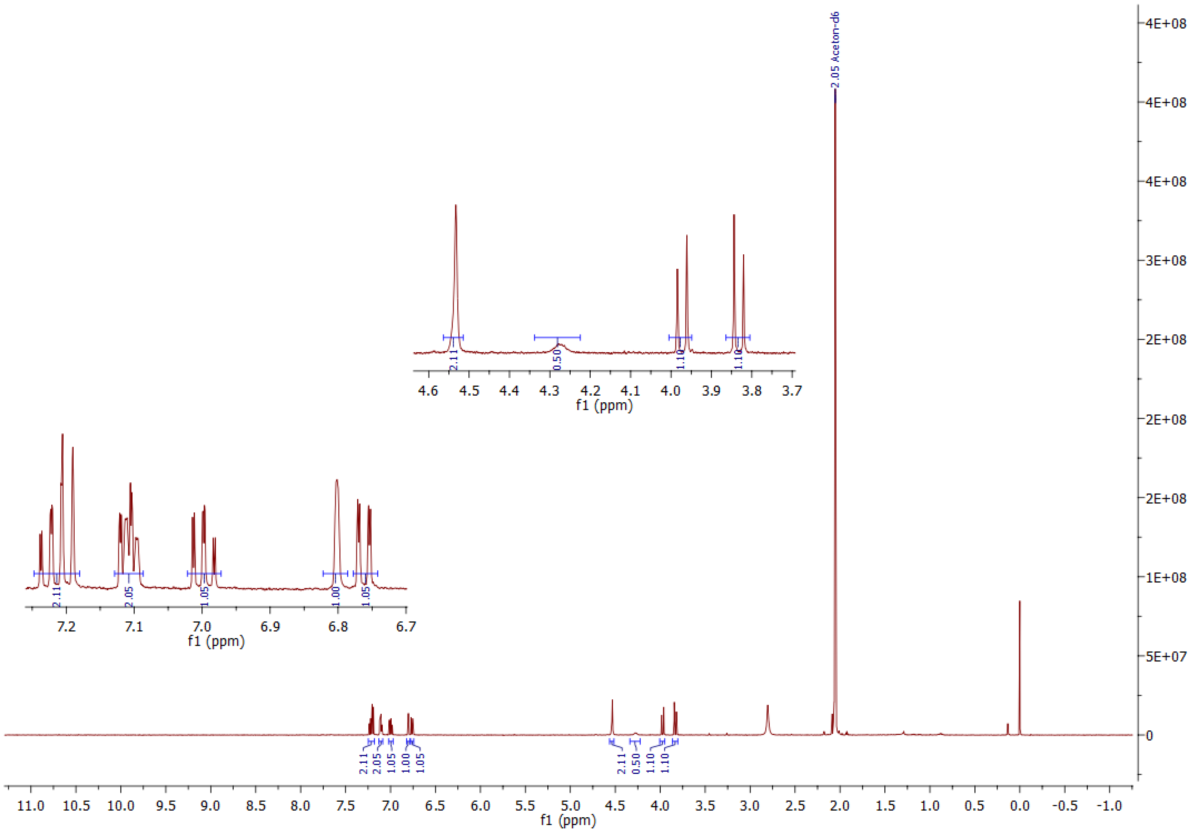

Figure S23: ^{1}H NMR spectrum of compound **5** measured in deuterated acetone.

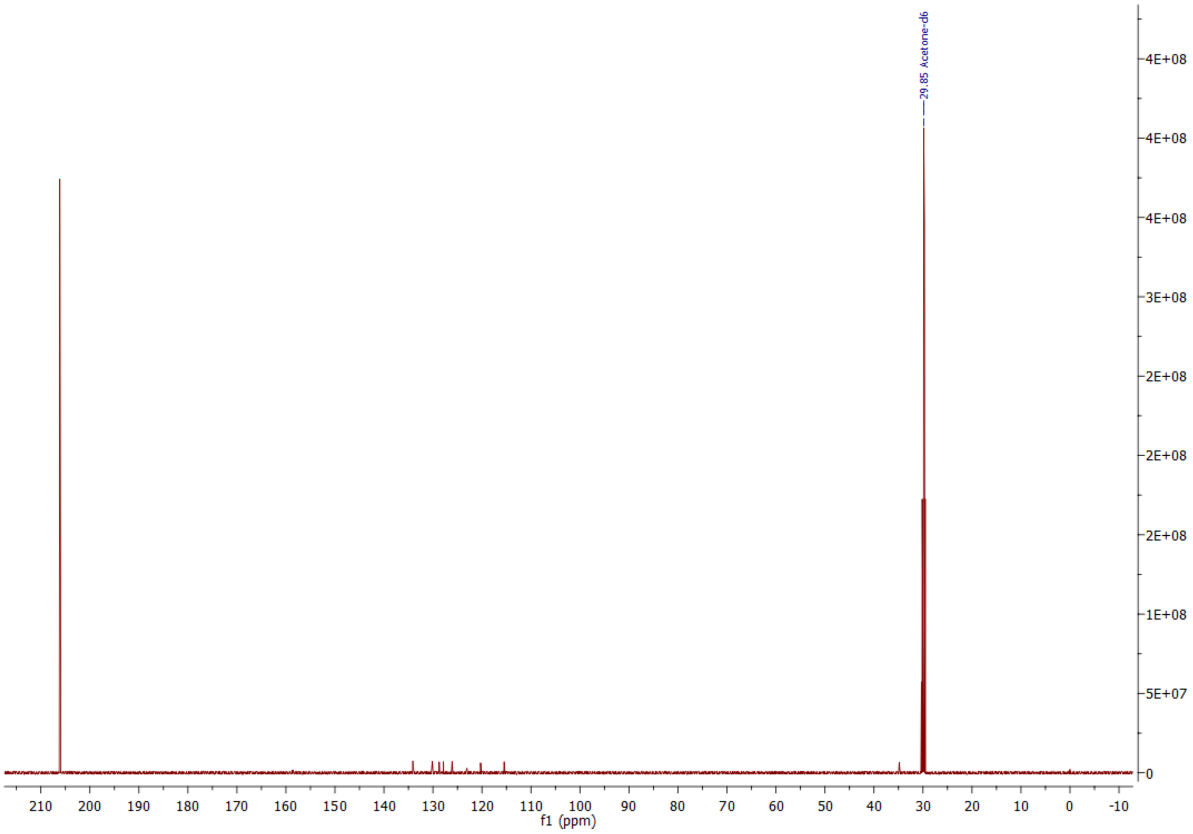

Figure S24: ^{13}C NMR spectrum of compound **5** measured in deuterated acetone.

III. 6-chlorobenzo[*c*]isothiazole (19)

In the reductive azo coupling of the halogenated S-diazocines the respective isothiazoles were formed as a byproduct. For the chloro-functionalized S-diazocine **1** the byproduct **19** could be isolated and fully characterized, which is in agreement with literature [2] [3].

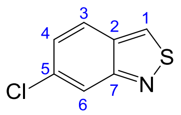

A colorless solid was obtained via sublimation.

melting point: 68 °C.

^{1}**H-NMR** (500 MHz, $CDCl_3$): δ = 9.12 (d, ^4J = 1.1 Hz, 1 H, *H*-1), 7.64 (m_c, 1 H, *H*-6), 7.54 (dd, ^3J = 9.2 Hz, ^5J = 0.8 Hz, 1 H, *H*-3), 6.97 (dd, ^3J = 9.2 Hz, ^4J = 1.6 Hz, 1 H, *H*-4) ppm.

^{13}**C-NMR** (125 MHz, $CDCl_3$): δ = 156.2 (*C*-1), 155.1 (*C*-7), 137.2 (*C*-5), 126.0 (*C*-4), 121.0 (*C*-3), 116.7 (*C*-2), 113.7 (*C*-6) ppm.

IR (ATR): ύ = 3109 (w), 1639 (m), 1547 (w), 1506 (w), 1459 (m), 1375 (m), 1290 (w), 1231 (w), 1112 (m), 1042 (m), 917 (m), 853 (m), 842 (m), 827 (m), 777 (s), 751 (w), 733 (m), 690 (w), 602 (w), 576 (s), 428 (vs), 402 (m) cm^{-1}.

MS (EI, 70 eV): m/z (%) = 169 (16) $[M+1]^+$, 135 (49) $[M-Cl]^+$.

MS (EI, HR, 70 eV): C_7H_4ClNS, m/z = calc.: 169.9747, found: 169.9743.

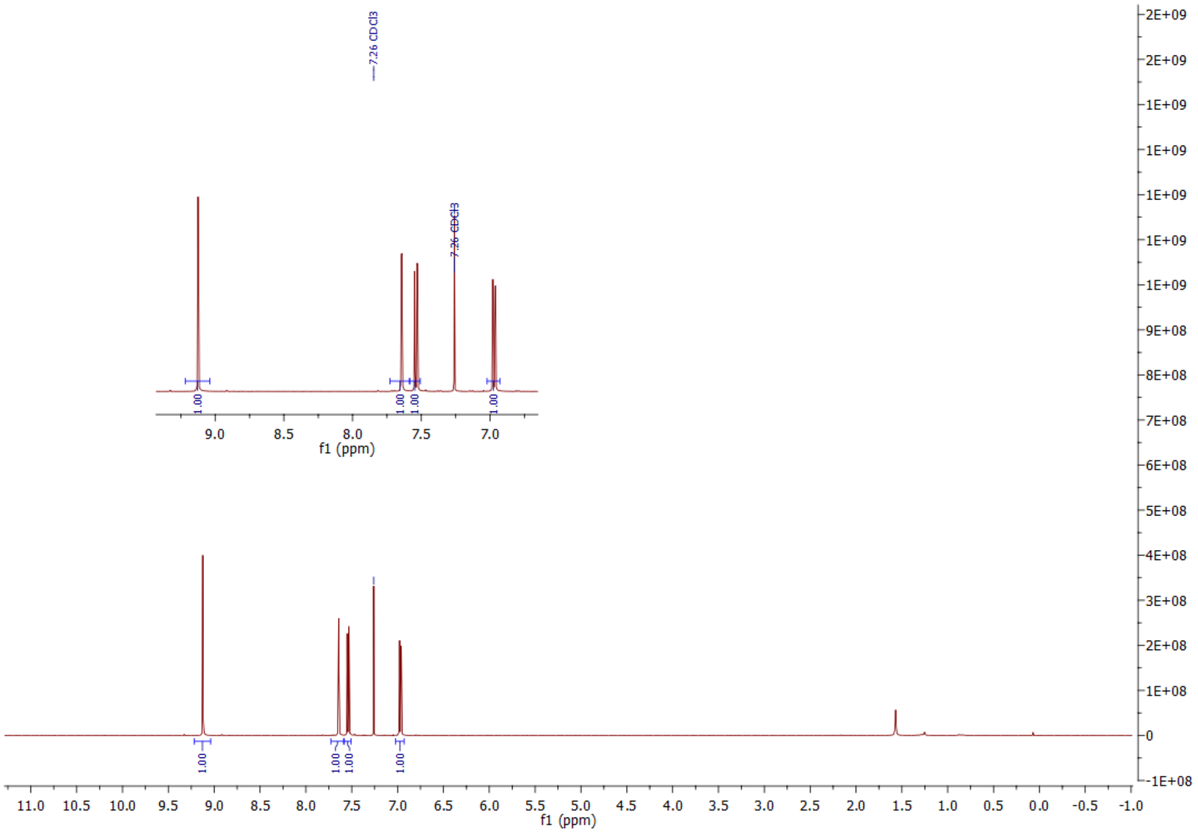

Figure S25: ^{1}H NMR spectrum of compound **19** measured in deuterated chloroform.

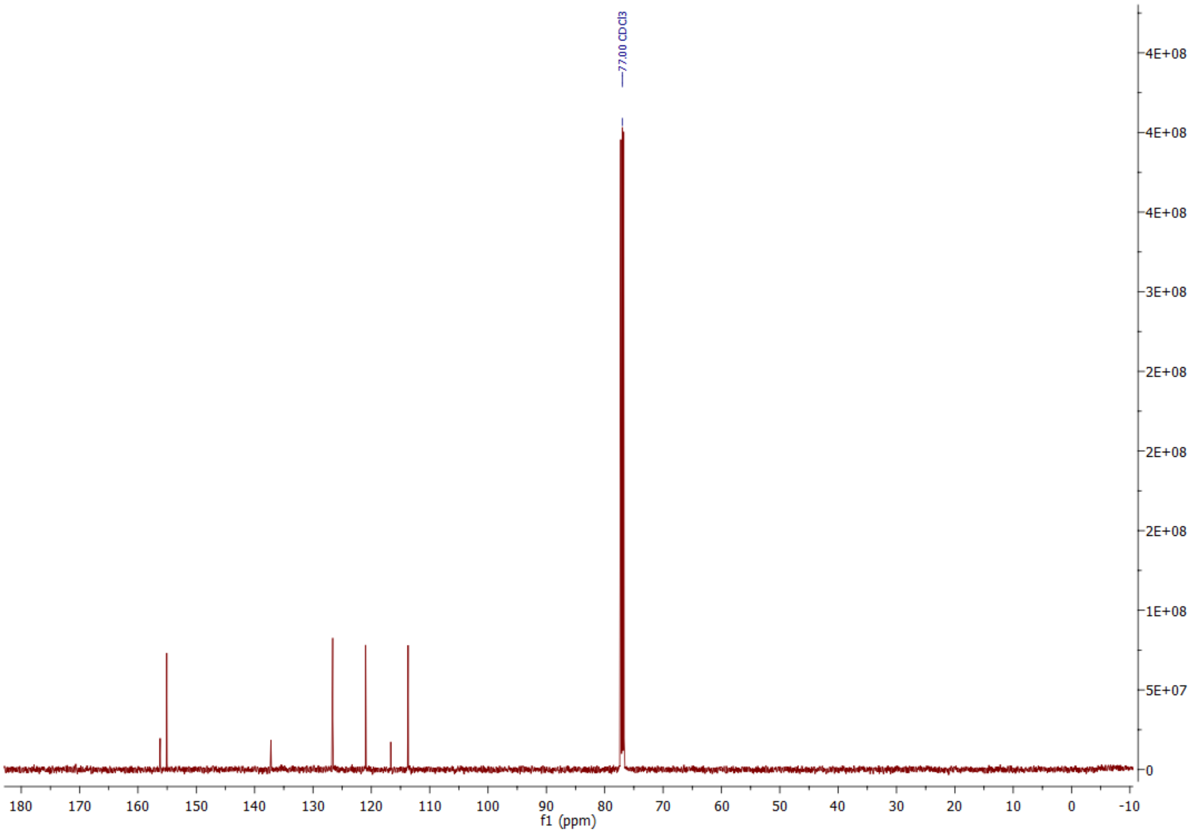

Figure S26: ^{13}C NMR spectrum of compound **19** measured in deuterated chloroform.

VI. UV–vis switching experiments

A solution of the respective S-diazocine in acetone **1**, **2**, **3**, **4** or **5** was prepared in the dark and the UV–vis *cis* spectra (black) were recorded. Then the solution was irradiated with 405 nm for 30 seconds and the UV–vis PSS spectra (red) were recorded.

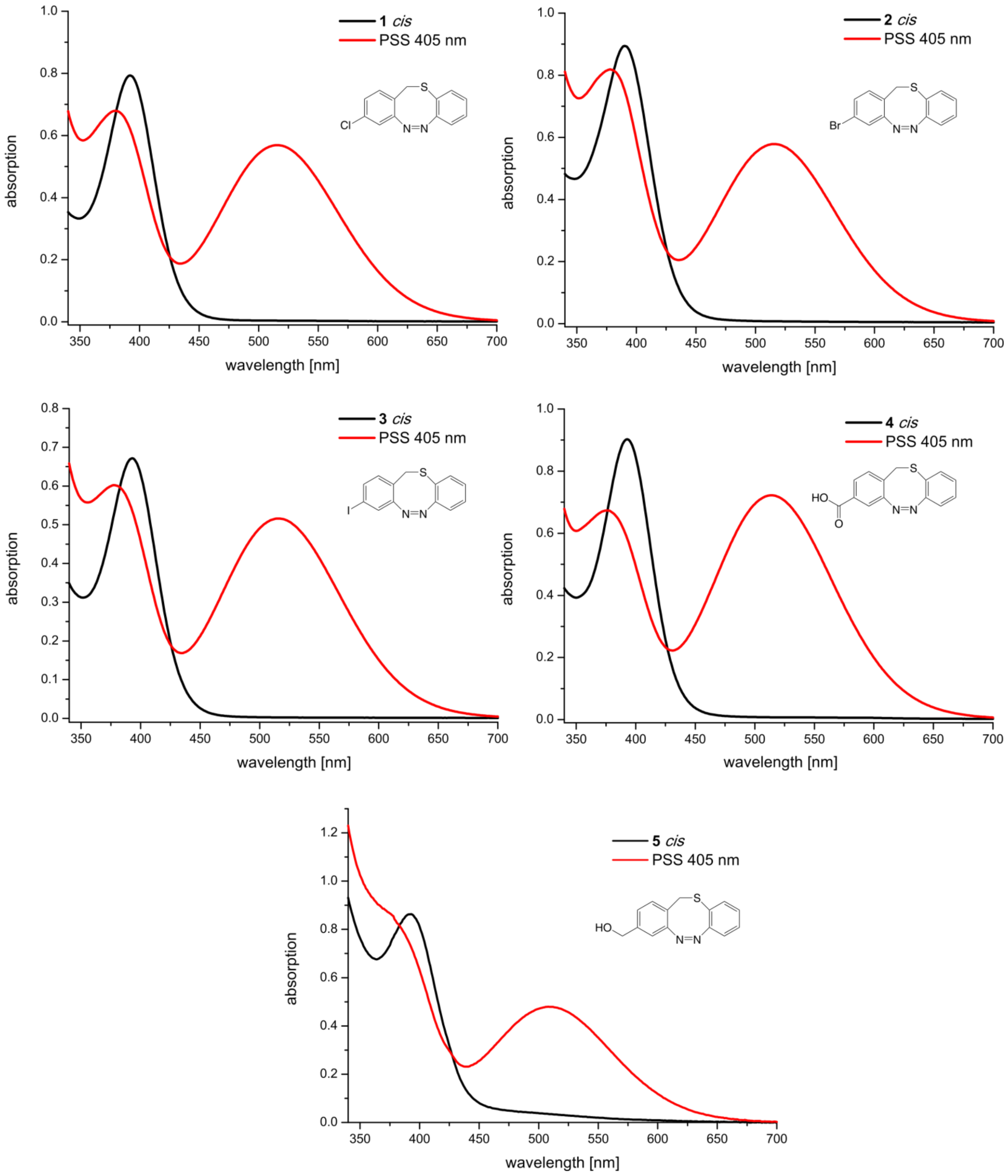

Figure S27: UV–vis spectra measured at 24 °C of the functionalized S-diazocines **1**, **2**, **3**, **4** and **5**; the *cis* spectra is plotted in black and the spectra of the PSS between *cis*/*trans* in red.

V. Crystallographic data for compound 3

Table S1. Crystal data and structure refinement for compound 3

Identification code	herges128	
Empirical formula	$C_{13}H_9IN_2S$	
Formula weight	352.18	
Temperature	200(2) K	
Wavelength	0.71073 Å	
Crystal system	Monoclinic	
Space group	$P2_1/c$	
Unit cell dimensions	a = 11.2206(5) Å	α = 90°.
	b = 12.7576(4) Å	β = 101.042(4)°.
	c = 8.7584(4) Å	γ = 90°.
Volume	1230.54(9) $Å^3$	
Z	4	
Density (calculated)	1.901 Mg/m^3	
Absorption coefficient	2.749 mm^{-1}	
F(000)	680	
Crystal size	0.08 x 0.12 x 0.17 mm^3	
Theta range for data collection	1.849 to 27.004°.	
Index ranges	-14<=h<=14, -16<=k<=16, -11<=l<=11	
Reflections collected	14765	
Independent reflections	2689 [R(int) = 0.0231]	
Completeness to theta = 25.242°	99.9 %	
Refinement method	Full-matrix least-squares on F^2	
Data / restraints / parameters	2689 / 0 / 155	
Goodness-of-fit on F^2	1.055	
Final R indices [I>2sigma(I)]	R1 = 0.0273, wR2 = 0.0759	
R indices (all data)	R1 = 0.0313, wR2 = 0.0778	
Extinction coefficient	0.0078(8)	
Largest diff. peak and hole	0.461 and -0.742 e.$Å^{-3}$	

Comments

A numerical absorption correction was performed (Tmin(max: 0.5901/0.7425). All non-hydrogen atoms were refined anisotropic. The C-H H atoms were located in difference map but were positioned with idealized geometry and refined isotropic with $U_{iso}(H) = 1.2\ U_{eq}(C)$ using a riding model.

Table S2. Atomic coordinates (x 10^4) and equivalent isotropic displacement parameters ($Å^2$x 10^3. U(eq) is defined as one third of the trace of the orthogonalized U^{ij} tensor.

	x	y	z	U(eq)
I(1)	8964(1)	7420(1)	3983(1)	38(1)
S(1)	7458(1)	3718(1)	9814(1)	42(1)
N(1)	6248(2)	4324(2)	5881(2)	32(1)
N(2)	5558(2)	4458(2)	6829(2)	32(1)
C(1)	7401(2)	4861(2)	6132(3)	28(1)
C(2)	7555(2)	5704(2)	5193(3)	29(1)
C(3)	8698(2)	6160(2)	5384(3)	29(1)
C(4)	9660(2)	5780(2)	6483(3)	33(1)
C(5)	9483(2)	4936(2)	7395(3)	32(1)
C(6)	8356(2)	4444(2)	7221(3)	30(1)
C(7)	8149(3)	3487(2)	8115(3)	35(1)
C(8)	6696(2)	4928(2)	9469(3)	30(1)
C(9)	5893(2)	5191(2)	8101(3)	28(1)
C(10)	5273(2)	6144(2)	7968(3)	34(1)
C(11)	5455(3)	6837(2)	9199(3)	37(1)
C(12)	6230(3)	6578(2)	10569(3)	37(1)
C(13)	6848(2)	5641(2)	10700(3)	34(1)

Table S3. Anisotropic displacement parameters ($Å^2$x 10^3). The anisotropic displacement factor exponent takes the form: -2□^{2}[h^2 $a^{*2}U^{11}$ + ... + 2 h k a* b* U^{12}]

	U^{11}	U^{22}	U^{33}	U^{23}	U^{13}	U^{12}
I(1)	40(1)	38(1)	36(1)	2(1)	9(1)	-2(1)
S(1)	54(1)	36(1)	39(1)	14(1)	16(1)	14(1)
N(1)	33(1)	31(1)	29(1)	0(1)	0(1)	-4(1)
N(2)	31(1)	30(1)	33(1)	0(1)	0(1)	-4(1)
C(1)	30(1)	28(1)	27(1)	-5(1)	4(1)	1(1)
C(2)	29(1)	30(1)	27(1)	-2(1)	3(1)	2(1)
C(3)	34(1)	28(1)	26(1)	-1(1)	9(1)	0(1)
C(4)	27(1)	39(1)	31(1)	-4(1)	5(1)	-1(1)
C(5)	31(1)	36(1)	29(1)	-2(1)	4(1)	8(1)
C(6)	33(1)	29(1)	28(1)	-3(1)	5(1)	4(1)
C(7)	41(1)	27(1)	38(1)	3(1)	7(1)	8(1)
C(8)	29(1)	30(1)	32(1)	2(1)	9(1)	-2(1)
C(9)	25(1)	26(1)	33(1)	1(1)	6(1)	-5(1)
C(10)	30(1)	32(1)	41(1)	6(1)	4(1)	-1(1)
C(11)	35(1)	29(1)	50(2)	1(1)	14(1)	1(1)
C(12)	40(1)	35(1)	40(1)	-8(1)	16(1)	-3(1)
C(13)	34(1)	39(1)	30(1)	1(1)	7(1)	-1(1)

Table S4. Hydrogen coordinates (x 10^4) and isotropic displacement parameters ($Å^2$x 10 ^{3}).

	x	y	z	U(eq)
H(2)	6898	5963	4439	35
H(4)	10436	6102	6605	39
H(5)	10141	4685	8155	39
H(7A)	8938	3131	8463	43
H(7B)	7620	3002	7404	43
H(10)	4727	6315	7032	41
H(11)	5046	7493	9104	45
H(12)	6337	7049	11425	45
H(13)	7387	5476	11644	41

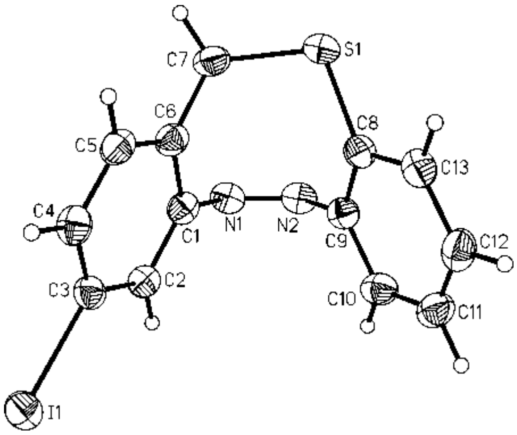

Table S5. Bond lengths [Å] and angles [°].

I(1)-C(3)	2.079(2)	C(4)-C(5)	1.378(4)
S(1)-C(8)	1.763(3)	C(5)-C(6)	1.394(4)
S(1)-C(7)	1.829(3)	C(6)-C(7)	1.493(4)
N(1)-N(2)	1.250(3)	C(8)-C(9)	1.395(3)
N(1)-C(1)	1.444(3)	C(8)-C(13)	1.396(4)
N(2)-C(9)	1.448(3)	C(9)-C(10)	1.394(3)
C(1)-C(2)	1.385(3)	C(10)-C(11)	1.379(4)
C(1)-C(6)	1.396(3)	C(11)-C(12)	1.381(4)
C(2)-C(3)	1.388(4)	C(12)-C(13)	1.376(4)
C(3)-C(4)	1.388(3)		
C(8)-S(1)-C(7)	105.57(12)	C(5)-C(6)-C(7)	122.7(2)
N(2)-N(1)-C(1)	119.0(2)	C(1)-C(6)-C(7)	119.5(2)
N(1)-N(2)-C(9)	119.7(2)	C(6)-C(7)-S(1)	115.34(18)
C(2)-C(1)-C(6)	122.3(2)	C(9)-C(8)-C(13)	118.0(2)
C(2)-C(1)-N(1)	119.2(2)	C(9)-C(8)-S(1)	124.79(19)
C(6)-C(1)-N(1)	118.2(2)	C(13)-C(8)-S(1)	117.04(19)
C(1)-C(2)-C(3)	118.1(2)	C(10)-C(9)-C(8)	120.8(2)
C(2)-C(3)-C(4)	121.0(2)	C(10)-C(9)-N(2)	116.6(2)
C(2)-C(3)-I(1)	119.12(18)	C(8)-C(9)-N(2)	122.3(2)
C(4)-C(3)-I(1)	119.87(19)	C(11)-C(10)-C(9)	119.8(2)
C(5)-C(4)-C(3)	119.7(2)	C(10)-C(11)-C(12)	120.0(2)
C(4)-C(5)-C(6)	121.1(2)	C(13)-C(12)-C(11)	120.2(3)
C(5)-C(6)-C(1)	117.7(2)	C(12)-C(13)-C(8)	121.1(2)

[1] Fulmer, G. R.; Miller, A. J. M.; Sherden, N. H.; Gottlieb, H. E.; Nudelman, A.; Stoltz, B. M.; Bercaw, J. E.; Goldberg, K. I. *Organometallics* **2010**, *29*, 2176-2179.

[2] Singerman, G. M. *Journal of Heterocyclic Chemistry* **1975**, *12*, 877-882.

[3] Gulf Research and Develop. Comp. - US3890340 **1975**.
